# Discovery of Amide-Functionalized Benzimidazolium Salts as Potent α-Glucosidase Inhibitors

**DOI:** 10.3390/molecules26164760

**Published:** 2021-08-06

**Authors:** Imran Ahmad Khan, Matloob Ahmad, Usman Ali Ashfaq, Sadia Sultan, Magdi E.A. Zaki

**Affiliations:** 1 Department of Chemistry, Government College University, Faisalabad 38000, Pakistan; imrankhan707470@gmail.com; 2 Department of Bioinformatics and Biotechnology, Government College University, Faisalabad 38000, Pakistan; ashfaqua@gcuf.edu.pk; 3 Faculty of Pharmacy, Universiti Teknologi MARA, Puncak Alam Campus, Bandar Puncak Alam 42300, Selangor Darul Ehsan, Malaysia; 4 Atta-ur-Rahman Institute for Natural Products Discovery (AuRIns), Universiti Teknologi MARA, Puncak Alam Campus, Bandar Puncak Alam 42300, Selangor Darul Ehsan, Malaysia; 5 Department of Chemistry, Faculty of Science, Imam Mohammad Ibn Saud Islamic University (IMSIU), Riyadh 11623, Saudi Arabia

**Keywords:** benzimidazole derivatives, benzimidazolium salts, molecular docking, anti-diabetic studies, α-glucosidase inhibition

## Abstract

α-Glucosidase inhibitors (AGIs) are used as medicines for the treatment of diabetes mellitus. The α-Glucosidase enzyme is present in the small intestine and is responsible for the breakdown of carbohydrates into sugars. The process results in an increase in blood sugar levels. AGIs slow down the digestion of carbohydrates that is helpful in controlling the sugar levels in the blood after meals. Among heterocyclic compounds, benzimidazole moiety is recognized as a potent bioactive scaffold for its wide range of biologically active derivatives. The aim of this study is to explore the α-glucosidase inhibition ability of benzimidazolium salts. In this study, two novel series of benzimidazolium salts, i.e., 1-benzyl-3-{2-(substituted) amino-2-oxoethyl}-1*H*-benzo[*d*]imidazol-3-ium bromide **9a**–**m** and 1-benzyl-3-{2-substituted) amino-2-oxoethyl}-2-methyl-1*H*-benzo[*d*] imidazol-3-ium bromide **10a**–**m** were screened for their in vitro α-glucosidase inhibitory potential. These compounds were synthesized through a multistep procedure and were characterized by ^1^*H*-NMR, ^13^C-NMR, and EI-MS techniques. Compound **10d** was identified as the potent α-glucosidase inhibitor among the series with an IC_50_ value of 14 ± 0.013 μM, which is 4-fold higher than the standard drug, acarbose. In addition, compounds **10a**, **10e**, **10h**, **10g**, **10k**, **10l**, and **10m** also exhibited pronounced potential for α-glucosidase inhibition with IC_50_ value ranging from 15 ± 0.037 to 32.27 ± 0.050 µM when compared with the reference drug acarbose (IC_50_ = 58.8 ± 0.12 μM). A molecular docking study was performed to rationalize the binding interactions of potent inhibitors with the active site of the α-glucosidase enzyme.

## 1. Introduction

The benzimidazole ring system has established its reputation amongst heterocyclic templates for its biologically active derivatives. Benzimidazole derivatives have displayed exceptional clinical and biological applications, such as anticancer [1], antifungal [2], antimalarial [3], antibacterial [4], anti-HIV [5], anti-inflammatory [6], antioxidant [7], antiprotozoal [8], and enzyme inhibitory activities [9]. In addition, it serves as an essential component of various commercially available biocides [10,11].

**Figure 1:**
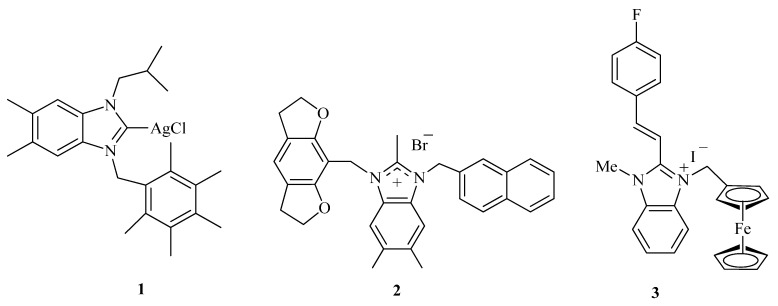
The chemical structures of biologically active benzimidazolium salts.

Benzimidazolium salts (derived from benzimidazole) have been extensively explored the for design and discovery of new drugs [12,13,14]. Particularly, in organometallic chemistry, benzimidazolium salts have emerged as an influential class of ligands due to strong σ-donor and weak π-accepting ability. Besides multiple synthetic and catalytic applications, benzimidazolium salts are also proven as effective antibacterial **1** [15], anticancer **2** [16], antimalarial, and anti-leishmanial agents **3** [17,18] (Figure 1).

Diabetes mellitus is a prevailing disorder, arisen due to a high level of blood glucose. The α-Glucosidase enzyme is responsible for the lysis of α-glucopyranoside bonds in disaccharides and oligosaccharides to form monosaccharides, ultimately regulating the blood glucose availability and postprandial hyperglycemia in type-2 diabetic patients [19]. The role of α-glucosidase inhibitors is to reduce hyperglycemia, thus considered as first-line sugar-lowering agents [20]. At present, a number of α-glucosidase inhibitors such as acarbose, voglibose, and miglitol have been discovered [21]. These α-glucosidase inhibitors are capable to interrupt the hydrolysis of carbohydrates and thus, reduce postprandial blood glucose levels [22,23]. However, continuous intake of these drugs may cause severe side effects such as diarrhea, body pain, and gastrointestinal disorders [24,25]. Subsequently, the development of new α-glucosidase inhibitors associated with minimum side effects is the need of the time.

Over the recent years, heterocyclic compounds have been extensively studied as α-glucosidase inhibitors [26,27,28]. More specifically, the benzimidazole ring system has emerged as an efficient template for new and potential anti-diabetic agents [29,30,31]. Benzimidazolium salts having α-glucosidase inhibitory potential have appeared in a few literature reports [32]. To the best of our knowledge, this research work is among the few successful efforts to explore benzimidazolium salts as α-glucosidase inhibitors. Keeping in view with evidence from the literature for the biological activities of benzimidazolium salts, we have synthesized a library of novel 1,3-disubstituted benzimidazolium salts and have screened them for α-glucosidase inhibition studies. Furthermore, the structure-activity relationship has been established with the help of molecular docking studies.

## 2. Results and Discussion

### 2.1. Chemistry

**Scheme 1:**
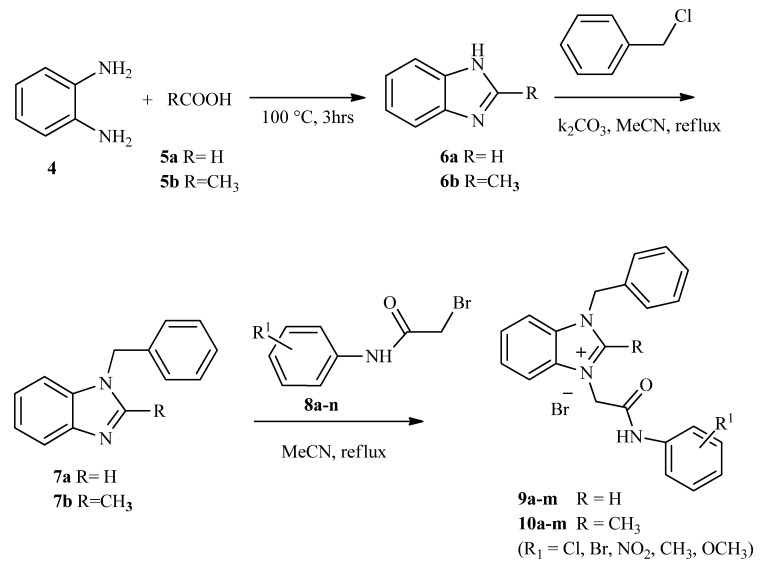
The lay-out for the synthesis of targeted benzimidazolium salts.

Twenty-six benzimidazolium salts **9**(**a**–**m**) and **10**(**a**–**m**) bearing amide functionality have been synthesized by following the methodology as illustrated in Scheme 1. It involves the reaction of *o*-phenylenediamine and respective carboxylic acid for 3 to 5 h to give 1*H*-benzimidazole and 2-methyl-1*H*-benzimidazole. These precursor benzimidazoles were treated with benzyl chloride in the presence of a base and using acetonitrile as a solvent to form corresponding *N*-benzylatedbenzimidazoles in 65–85% yields. Quaternization of the synthesized *N*-benzylbenzimidazole precursors with a range of substituted α-bromoacetamides resulted in the formation of targeted benzimidazolium salts **9**(**a**–**m**) and **10**(**a**–**m**). The chemical reaction was monitored by TLC using chloroform: methanol (4:1) mixture as a mobile phase. After completion of the reaction, products were filtered, washed, and purified using column chromatography.

### 2.2. Characterization

The formation of benzimidazolium salts was confirmed by ^1^*H*-NMR, ^13^C-NMR, and mass spectrometry (Supplementary Materials File). A characteristic sharp singlet between 9.91 and 10.3 ppm is observed for NC*H*N protons (imidazole ring) of compounds **9a**–**m**. For all benzimidazolium salts, protons of the methylene group attached to phenyl (–C*H*_2_–Ph) resonated at 5.51–5.86 ppm as a sharp singlet whereas the occurrence of another slightly downfield singlet in the range of 5.66–6.00 ppm is integrated for two protons of methylene linker of acetamide moiety. The acetanilide N*H* in benzimidazolium bromides appeared as an isolated singlet at 10.0–11.78 ppm. In ^1^*H*-NMR spectra of **9i**, **9j**, **9k**, **10i**, **10j**, and **10k**, a singlet peak in the range of 2.25–2.30 ppm confirmed the presence of methyl substituent. Similarly, the appearance of a singlet at 3.71–3.91ppm corresponded to the methoxy group of compounds **9l**, **9m**, **10l**, and **10m**. Aromatic protons of benzimidazolium salts **9**(**a**–**m**) and **10**(**a**–**m**) were detected in the range of 6.90–8.61ppm. In the case of ^13^*C*-NMR spectra, signals of methylene carbons and carbonyl carbons were observed in the range of 49–52 ppm and 162.6–166 ppm respectively. Furthermore, mass spectrometry data also confirmed the formation of title compounds. The NMR data of similar functionalities in the literature [33,34] was found supportive of our findings.

### 2.3. In Silico Studies (Molecular Docking)

**Figure 2:**
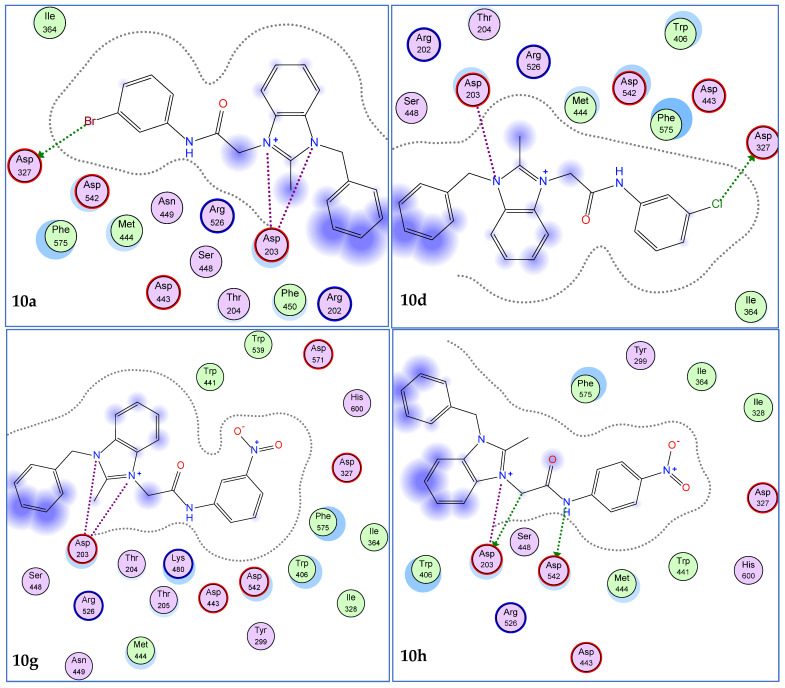

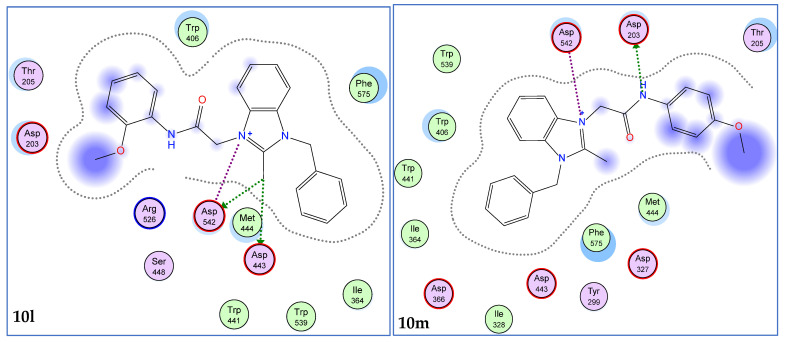
2D interaction modes of active compounds (**10a**, **10d**, **10g**, **10h**, **10l**, and **10m**). The blue color indicates ligand exposure and the green color dotted lines indicates the interactions of receptor enzyme with ligands.

**Figure 3:**
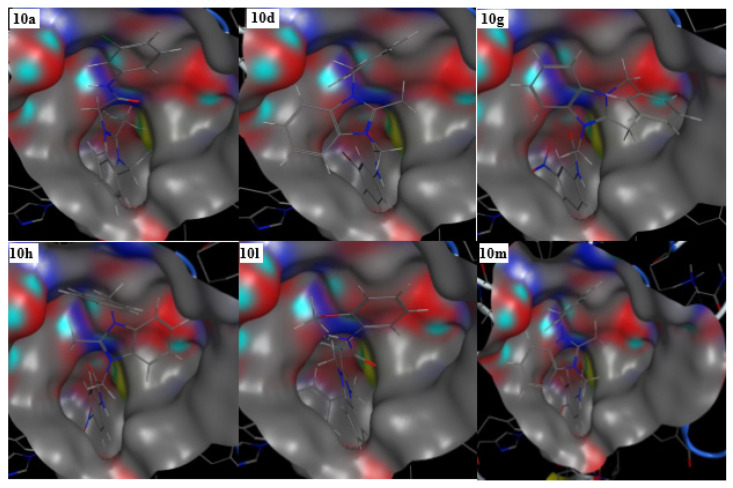
3D interaction modes of active compounds (**10a**, **10d**, **10g**, **10h**, **10l**, and **10m**). Color portion shows the pocket. Selected and red portions indicate the ligand exposure points.

The in silico techniques have established their reputation in the field of drug discovery. The study is helpful in identifying the potential biologically active compounds as the targets for synthesis, thus minimizing the hardships of laborious synthetic work. Considering the effectiveness of this tool, we executed the molecular docking studies of a library of benzimidazolium salts. Molecular docking was performed with the help of the MOE software to screen out the compounds having the best residual interactions with the receptor protein. Compounds were ranked on the basis of docking scores, rmsd values, and interactions with the selected pocket of the receptor enzyme. Results of molecular docking are presented in Figure 2 and Figure 3 and Table 1.

Among the screened derivatives, the benzimidazolium salts **10a**–**m** showed good docking scores and significant interactions with the selected pocket of the receptor protein in comparison to the benzimidazolium salts **9a**–**m** (Table 1, Figure 2). The binding energy values of most of these derivatives were found close to the standard drug, acarbose (−16.18 Kcal/mol). These ligands also occupied the selected pocket of the receptor enzyme effectively, as shown in 3D maps (Figure 3).

Most of the ligands interacted with Asp203, Asp327, and Asp542 residues among the reference/selected residues (Asp203, Asp542, Asp327, His600, Arg526) as revealed by the 2D maps and were considered responsible for the inhibition of the α-glucosidase enzyme (Figure 2). Nakamura and group had also observed the interactions of Asp203, Asp327, and Asp542 residues with the salacinol and its derivatives during in silico screening against α-glucosidase enzyme [35]. Similarly, the aglycone of curculigoside A, and derivatives, blocked the activity of α-glucosidase enzyme via bindings with Asp203, Asp327, and Asp542 residues [36]. In silico analysis of anthocyanidins and anthocyanins proved them good inhibitors of the α-glucosidase enzyme. These compounds also exhibited inhibitory action by interacting with the selected residues, Asp203, Asp327, and Asp542 [37].

**Figure 4:**
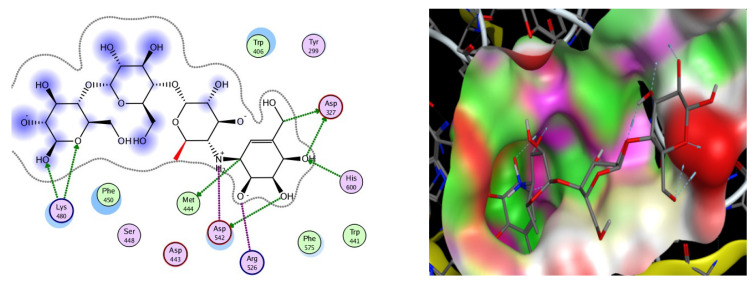
2D (**Left**) and 3D (**Right**) interaction modes of the standard drug, acarbose.

Prominently, the derivatives **10a**, **10d**, **10g**, **10h**, **10l**, and **10m** exhibited best docking scores (−10.06 to −11.15 Kcal/mol) and rmsd values less than 2Å (Table 1). Regarding the interaction modes, the compounds **10a** and **10d** showed interactions with the common residues, Asp203 and Asp327 (Figure. 2). The docking scores for these ligands were found to be −10.87 and −10.85 Kcal/mol, respectively. Similarly, the ligands **10h** and **10m** also exhibited good docking scores (−10.94 and −13.43 Kcal/mol) and bindings with common residues, Asp203 and Asp542 (Figure 2). The ligands **10g** and **10m** showed interactions with Asp203 and Asp542, respectively. In order to check the validity of docking experiments, acarbose was also docked into the selected pocket of the enzyme and it showed binding interactions with selected residues, His600, Asp542, Arg526, and Asp327 (Figure 4). It is important to note that the presence of methyl, bromo, chloro, methylene, acetamide groups, and imidazole ring were found indispensable for the in silico inhibition of the α-glucosidase enzyme.

### 2.4. In Vitro α-Glucosidase Inhibitory Activity

Benzimidazole derivatives have been reported as excellent inhibitors for α-glucosidase, dipeptidyl Peptidase IV, aldose reductase, and diacylglycerol acyltransferase [38,39]. In this study, two series of benzimidazolium salts that are quaternary derivatives of benzimidazole have been synthesized in search of potent α-glucosidase inhibitors. The compounds **9**(**a**–**m**) and **10**(**a**–**m**) were screened for in vitro α-glucosidase inhibitory activity according to reported protocol by using acrobose as a reference drug. The synthesized benzimidazolium salts exhibited a varying degree of α-glucosidase inhibitory potential with IC_50_ values ranging from 14 to 279.28 µM presented in Table 2. The structure–activity relationship established the role of various substituents on phenyl rings and their positions with biological activities. First of all, compounds **9a**–**m** were found to be inactive or weakly active against the enzyme, while compounds having C^2^-substituted benzimidazolium salts **10a**–**m** demonstrated excellent α-glucosidase inhibitory profile (Table 2). These results strengthen the fact that C^2^-substituted benzimidazole variants are more susceptible to α-glucosidase inhibition. Recently, Mentes and his coworkers synthesized 4-(5-fluoro-2-substituted-1*H*-benzimidazol-6-yl)morpholine derivatives and indicated that C^2^ substitution of benzimidazole has a significant effect on α-glucosidase inhibitory activities [40]. The strong impact of C^2^ substituted benzimidazole on α-glucosidase and urease inhibitory activities is observed by other researchers too [41]. Predominately, compound **10d** (1-benzyl-3-(2-((3-bromophenyl)amino)-2-oxoethyl)-2-methyl-1*H*-benzo[*d*]imidazole-3-ium bromide) exhibited the highest α-glucosidase inhibitory potential with IC_50_ value 14 + 0.013 µM, which is 4-fold higher than reference drug. Compounds **10m**, **10h**, **10g**, **10a**, **10l**, **10e**, and **10k** exhibited better activity than acarbose with an IC_50_ value of 16.16 ± 0.026, 17 ± 0.067, 19 ± 0.086, 22 ± 0.040, 25 ± 0.011, 30 ± 0.049 and 32.27 ± 0.050 µM, respectively.

Compounds **10c**, **10d**, **10e** having ortho, meta, and para-substituted bromo group on phenyl rings, showed higher activity than acarbose (standard drug). The shifting of bromo from position 2 to position 3 led to a dramatic increase in activity. Among all of the halo substituted derivatives, **10d** was the most active with the highest α-glucosidase inhibitory potential. Replacing of the bromo substituent with the chloro substituent caused a slight decline in the α-glucosidase inhibitory potential for **10a** and **10b**, as the compound appeared as a moderate to poor inhibitor. It is reflected that nature and change in position of the halo-group have a notable effect on the α-glucosidase inhibitory activity. 

Among nitro-substituted compounds, **10g** and **10h**, having a substitution at the meta and para positions, demonstrated promising α-glucosidase inhibitory results due to a strong electron-withdrawing effect. However, **10f**, having nitro-substitution at the ortho position, showed considerable decline in activity.

**Figure 5:**
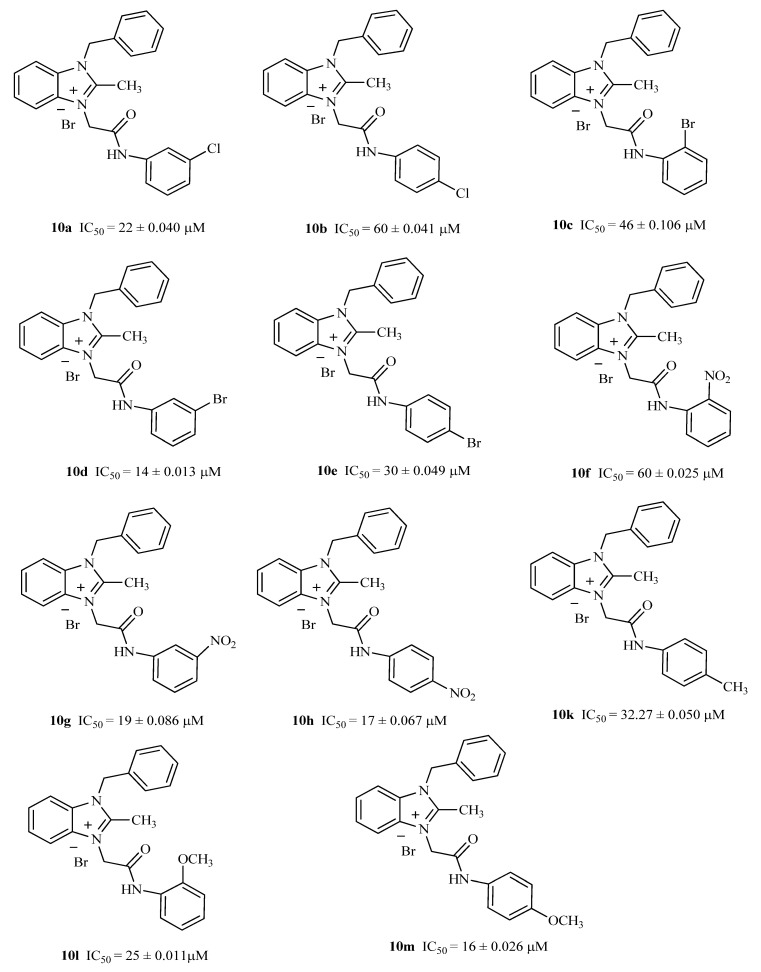
The chemical structures of potent α-glucosidase inhibitors.

The bioactivity results of **10l** and **10m** benzimidazolium salts with methoxy substitution at the ortho and para positions, also showed their effectiveness for α-glucosidase inhibition. Higher activities of these compounds containing an electron-donating methoxy group are due to resonance stabilization of the ortho and para positions. Contrary to these results, methyl-substituted derivatives **10i**–**k** showed varying degrees of α-glucosidase inhibitory potential. Compounds **10i** and **10j** containing methyl groups at the ortho and meta positions appeared as poor inhibitors, whereas, **10k** having methoxy at the para position was found to be moderately active (Figure 5).

It is clear from direct comparison of halo and methoxy/methyl-substituted compounds that there is a slight decrease in the α-glucosidase inhibitory profile for methoxy/methyl-substituted benzimidazolium salts. The decreasing order of α-glucosidase inhibitory potential of the active compounds can be listed as **10d** > **10m** > **10h** > **10g** > **10a** > **10l** > **10e** > **10k** > **10c** > acarbose. Compounds **9m** in the first series, and **10i** in the second series, having electron-donating substitution (methoxy and methyl), exhibited the lowest α-glucosidase inhibitory results in their respective series. It can be concluded from the above IC_50_ results that the position of both electron-donating and withdrawing groups on the phenyl ring have a vital role in determining the efficacy of α-glucosidase inhibition.

## 3. Materials and Methods

### 3.1. General

All chemicals and solvents used in this synthesis were purchased from Sigma-Aldrich (Munich, Germany) and Daejung Chemicals (Siheung-si, South Korea). Solvents such methanol, acetonitrile, and chloroform used in this synthesis were purified and dried by a solvents purification system. Melting points were recorded by using the Stuart SMP 40 melting point apparatus. Spectroscopic studies (^1^H and ^13^C-NMR) were carried out by using the Bruker 600 MHz spectrometer in DMSO-d_6_ solvent. 

### 3.2. General Procedure for the Preparation of Benzimidazole Precursors **6**(**a**–**b**)

*o*-Phenylenediamine **4** (5.4 g, 0.05 mol) and formic acid or acetic acid (0.075 mol) were mixed in a round bottom flask. The mixture was heated during stirring at 100 °C for 3 h. Afterwards, the contents of the flask were cooled to room temperature and an aqueous solution of sodium hydroxide (10% *w*/*v*) was added dropwise to attain a pH~8. The ppts. of the corresponding benzimidazole were formed, which were filtered, washed with ice-cold water, and dried. The products were purified by recrystallization from methanol.

*1H-Benzimidazole* (**6a**): Yield: 85%; m.p. 172 °C (Literature m.p. 171–173 °C [42]).
*2-Methyl-1H-Benzimidazole* (**6b**): Yield: 81%; m.p. 176 °C (Literature m.p. 176–178 °C [42]).

### 3.3. General Procedure for Preparation of N-Benzylatedbenzimidazoles **7**(**a**–**b**)

A mixture of benzimidazole **6a** or **6b** (0.05 mol), CTAB (0.05 mol), acetonitrile (30 mL), and K_2_CO_3_ (0.1 mol) was stirred at room temperature. Benzyl chloride (0.075 mol) was added drop-wise to the reaction mixture over a period of 5 min. Afterwards, after stirring, and the reaction mixture was subjected to reflux for 8 h. The progress of the reaction was monitored by using TLC in a mixture of chloroform and methanol in a 4:1 ratio as a mobile phase. After the completion of the reaction, the contents of the flask were cooled to room temperature and poured into crushed ice. The crude product thus obtained was purified by column chromatography.

*1-Benzyl-1H-benzimidazole* (**7a**): White crystalline solid; Yield: 85%; m.p. 118 °C (Literature m.p. 115–118 °C [43,44]); ^1^H-NMR (DMSO-d6, 600 MHz) δ (ppm): 5.60 (s, 2H, CH_2_-Ph), 7.10–7.22 (m, 3H, Ar-H), 7.30–7.35 (m, 5H, Ar-H), 7.75 (d, *J* = 7.8 Hz, 1H, Ar-H), 7.86 (s, 1H, CH=N). ^13^C-NMR (DMSO-d6, 150 MHz) δ (ppm): 48.8, 111.0, 121.5 (2C), 122.5, 124, 128 (2C), 128.5, 130, 134, 135.7, 144.1, 144.8 (C = N).
*1-Benzyl-2-methyl-1H-benzimidazole* (**7b**): Waxy solid; Yield: 65%; ^1^H-NMR (DMSO-d6, 600 MHz) δ (ppm): 2.45 (s, 3H, CH_3_), 5.30 (s, 2H, CH_2_-Ph), 7.10 (t, 3H, Ar-H), 7.35–7.42 (m, 5H, Ar-H), 7.70 (d, *J* = 7.2 Hz, 1H, Ar-H). ^13^C-NMR (DMSO-d6, 150 MHz) δ (ppm): 14.3, 46.4, 110.0, 121.5, 122.5 (2C), 124, 126 (2C), 127.5, 129, 133, 135.7, 141.2, 143.0 (C = N).

### 3.4. General Procedure for the Preparation of Benzimidazolium Salts **9**(**a**–**m**)

1-Benzyl-1*H*-benzimidazole **7a** (0.22 g, 1 mmol) was dissolved in acetonitrile (30 mL) in a round bottom flask. The solution of 2-bromo-*N*-arylacetamides **8**(**a**–**m**) (1 mmol) in acetonitrile (10 mL) was added dropwise in the round bottom flask and the reaction mixture was refluxed for 12–14 h. The completion of the reaction was observed by thin-layer chromatography. Finally, the contents were cooled in an ice bath to obtain the crude solid salts which were purified by column chromatography.

*1-Benzyl-3-[2-{(3-chlorophenyl)amino}-2-oxoethyl]-1H-benzo[d]imidazol-3-ium bromide* (**9a**): White crystalline solid, Yield: 80%; m.p. 205–207 °C. ^1^H-NMR (DMSO-d6, 600 MHz) δ (ppm): 5.63 (s, 2H, CH_2_-Ph), 5.89 (s, 2H, CH_2_-CO), 7.19 (d, 1H, *J* = 7.98 Hz, Ar-H), 7.41 (t, 2H, *J* = 8.07 Hz, Ar-H), 7.45 (t, 2H, *J* = 7.38 Hz, Ar-H), 7.51 (d, 1H, *J* = 8.28 Hz, Ar-H), 7.54 (d, 2H, *J* = 7.32 Hz, Ar-H), 7.67–7.72 (m, 2H, Ar-H), 7.81 (s, 1H, Ar-H), 8.01 (d, 1H, *J* = 7.68 Hz, Ar-H), 8.07 (d, 1H, *J* = 7.86 Hz, Ar-H), 9.97 (s, 1H, NCHN), 11.02 (s, 1H, NH). ^13^C-NMR (DMSO-d6, 150 MHz) δ (ppm): 49.2, 50.0, 113.8, 114.0, 117.7, 118.8, 123.8, 126.8, 127.0, 128.2 (2C), 128.4, 129.1(3C), 130.4, 130.7, 133.2, 133.7, 139.5, 143.5, 164.0 (C=O). MS (ESI+): *m*/*z* calcd for C_22_H_19_ClN_3_O^+^ [M − Br]^+^ 376; found 377 [M − Br + H]^+^.
*1-Benzyl-3-[2-{(4-chlorophenyl)amino}-2-oxoethyl]-1H-benzo[d]imidazol-3-ium bromide* (**9b**): White crystalline solid; Yield: 78%; m.p. 197–198 °C. ^1^H-NMR (DMSO-d6, 600 MHz) δ (ppm): 5.62 (s, 2H, CH_2_-Ph), 5.89 (s, 2H, CH_2_-CO), 7.39- 7.42 (m, 2H, Ar-H), 7.43–7.46 (m, 3H, Ar-H), 7.54 (d, 2H, *J* = 7.2 Hz, Ar-H), 7.65–7.67 (m, 3H, Ar-H), 7.68–7.71 (m, 1H, Ar-H), 8.01 (d, 1H, *J* = 6.72 Hz, Ar-H), 8.07 (d, 1H, *J* = 6.96 Hz, Ar-H), 9.98 (s, 1H, NCHN), 10.96 (s, 1H, NH). ^13^C-NMR (DMSO-d6, 150 MHz) δ (ppm): 49.2, 50.0, 113.8, 114.0, 121.0 (2C), 126.8, 127.0, 127.6, 128.2 (2C), 128.8 (2C), 129.0 (2C), 130.4, 132.0, 132.0, 133.7, 137.1, 143.6, 163.6 (C=O): MS (ESI+): *m*/*z* calcd for C_22_H_19_ClN_3_O^+^ [M − Br]^+^ 376; found 376 [M − Br]^+^.
*1-Benzyl-3-[2-{(2-bromophenyl)amino}-2-oxoethyl]-1H-benzo[d]imidazol-3-ium bromide* (**9c**): White crystalline solid; Yield: 78%; m.p. 192–194 °C. ^1^H-NMR (DMSO-d6, 600 MHz) δ (ppm): 5.68 (s, 2H, CH_2_-Ph), 5.87 (s, 2H, CH_2_-CO), 7.19 (t, 1H, *J* = 7.2 Hz, Ar-H), 7.39 (t, 2H, *J* = 7.2 Hz, Ar-H), 7.42 (q, 2H, *J* = 12.6 Hz, *J* = 14.4 Hz, Ar-H), 7.2 (t, 2H, *J* = 8.75 Hz, Ar-H) 7.60 (d, 1H, *J* = 7.26 Hz, Ar-H), 7.66–7.72 (m, 3H, Ar-H), 8.00 (d, 1H, *J* = 8.00 Hz, Ar-H), 8.06 (d, 1H, *J* = 8.06 Hz, Ar-H), 10.04 (s, 1H, NCHN), 10.50 (s, 1H, NH). ^13^C-NMR (DMSO-d6, 150 MHz) δ (ppm): 49.0, 50.0, 113.9 (2C), 126.8, 127.0, 127.5, 128.2 (5C), 128.8 (4C), 129.0, 131.0, 132.9, 133.8, 143.7, 164.1 (C=O). MS (ESI+): *m*/*z* calcd for C_22_H_19_BrN_3_O^+^ [M − Br]^+^ 420.0706; found 420.0714 [M − Br]^+^.
*1-Benzyl-3-[2-{(3-bromophenyl)amino}-2-oxoethyl]-1H-benzo[d]imidazol-3-ium bromide* (**9d**): White crystalline solid; Yield: 85%; m.p. 176–178 °C. ^1^H-NMR (DMSO-d6, 600 MHz) δ (ppm): 5.62 (s, 2H, CH_2_-Ph), 5.88 (s, 2H, CH_2_-CO), 7.31–7.36 (m, 2H, Ar-H), 7.42 (t, 1H, *J* = 8.46 Hz, Ar-H), 7.41–7.46 (m, 2H, Ar-H), 7.53–7.55 (m, 3H, Ar-H), 7.67–7.72 (m, 2H, Ar-H), 7.95 (t, 1H, *J* = 3.6 Hz, Ar-H), 8.01 (d, 1H, *J* = 6.93 Hz, Ar-H), 8.07 (d, 1H, *J* = 6.93 Hz, Ar-H), 9.96 (s, 1H, NCHN), 10.99 (s, 1H, NH).^13^C-NMR (DMSO-d6, 150 MHz) δ (ppm): 49.2, 50.0, 113.8, 114.0, 118.1, 121.6, 121.7, 126.7, 126.8, 127.0, 128.8, 128.2 (2C), 129.1 (2C), 130.4, 131.0, 132.0, 133.7, 139.7, 143.6, 163.9 (C=O). MS (ESI+): *m*/*z* calcd for C_22_H_19_BrN_3_O^+^ [M − Br]^+^ 420; found 420 [M − Br]^+^.
*1-Benzyl-3-[2-{(4-bromophenyl)amino)}-2-oxoethyl]-1H-benzo[d]imidazol-3-ium bromide* (**9e**): White crystalline solid; Yield: 75%; m.p. 150–152 °C. ^1^H-NMR (DMSO-d6, 600 MHz) δ (ppm): 5.60 (s, 2H, CH_2_-Ph), 5.88 (s, 2H, CH_2_-CO), 7.40 (t, 1H, *J* = 7.26 Hz, Ar-H), 7.45 (t, 2H, *J* = 7.35 Hz, Ar-H), 7.52–7.55 (m, 3H, Ar-H), 7.55 (t, 1H, *J* = 3.4 Hz, Ar-H), 7.6 (d, 2H, *J* =8.8 Hz, Ar-H), 7.76–7.71 (m, 2H, Ar-H), 8.0 (d, 1H, *J* =8.3 Hz, Ar-H), 8.06 (d, 1H, *J* =7.9 Hz, Ar-H), 9.95 (s, 1H, NCHN), 10.92 (s, 1H, NH). ^13^C-NMR (DMSO-d6, 150 MHz) δ (ppm): 49.2, 50.0, 113.8, 114.0, 115.7, 121.3, 126.8, 127.0, 128.2 (3C), 128.8, 129.1 (3C), 130.4, 131.8 (2C), 132.0, 133.7, 143.6, 144.20, 163.6 (C=O). MS (ESI+): *m*/*z* calcd for C_22_H_19_BrN_3_O^+^ [M − Br]^+^ 420; found 420 [M − Br]^+^.
*1-Benzyl-3-[2-{(2-nitrophenyl)amino}-2-oxoethyl]-1H-benzo[d]imidazol-3-ium bromide* (**9f**): White crystalline solid; Yield: 74%; m.p. 142–143 °C. ^1^H-NMR (DMSO-d6, 600 MHz) δ (ppm): 5.69 (s, 2H, CH_2_-Ph), 5.8 (s, 2H, CH_2_-CO), 7.38–7.41 (m, 1H, Ar-H), 7.44 (q, 3H, *J* = 7.4 Hz, Ar-H), 7.52 (d, 2H, *J* = 7.2 Hz, Ar-H), 7.67–7.73 (m, 3H, Ar-H), 7.77 (t, 1H, *J* = 7.8 Hz, Ar-H), 8.0 (t, 3H, *J* = 7.35 Hz, Ar-H), 9.99 (s, 1H, NCHN), 11.2 (s, 1H, NH). ^13^C-NMR (DMSO-d6, 150 MHz) δ (ppm): 49.0, 50.0, 113.8, 114.0, 121.0, 125.0 (2C), 126.8, 127.0, 128.2 (3C), 128.8, 129.0 (3C), 130.0, 132.0, 134.1, 142.8, 143.5, 164.2 (C=O). MS (ESI+): *m*/*z* calcd for C_22_H_19_N_4_O_3_^+^ [M − Br]^+^ 387; found 388 [M − Br + H]^+^.
*1-Benzyl-3-[2-{(3-nitrophenyl)amino}-2-oxoethyl]-1H-benzo[d]imidazol-3-ium bromide* (**9g**): White crystalline solid; Yield: 75%; m.p. 162–163 °C. ^1^H-NMR (DMSO-d6, 600 MHz) δ (ppm): 5.63 (s, 2H, CH_2_-Ph), 5.86 (s, 2H, CH_2_-CO), 7.38–7.41 (m, 1H, Ar-H), 7.44 (t, 2H, *J* = 14.7 Hz, Ar-H), 7.52 (d, 2H, *J* = 7.86 Hz, Ar- H), 7.66–7.71 (m, 3H, Ar-H), 7.90 (d, 1H, *J* = 7.68 Hz, Ar-H), 7.97–8.00 (m, 2H, Ar-H), 8.05 (d, 1H, *J* = 7.68 Hz, Ar-H), 8.61 (s, 1H, Ar-H), 9.93 (s, 1H, NCHN), 11.27 (s, 1H, NH). ^13^C-NMR (DMSO-d6, 150 MHz) δ (ppm): 49.2, 50.0, 113.4, 113.8, 114.0, 118.6, 125.3, 126.8, 127.0, 128.2 (2C), 128.87, 129.1 (2C), 130.4 (2C), 130.5, 132.0, 139.2, 143.5, 148.0, 164.3 (C=O). MS (ESI+): *m*/*z* calcd for C_22_H_19_N_4_O_3_^+^ [M − Br]^+^ 387; found 387 [M − Br]^+^.
*1-Benzyl-3-[2{(4-nitrophenyl)amino}-2-oxoethyl]-1H-benzo[d]imidazol-3-ium bromide* (**9h**): White crystalline solid; Yield: 70%; m.p. 152–153 °C. ^1^H-NMR (DMSO-d6, 600 MHz) δ (ppm): 5.64 (s, 2H, CH_2_-Ph), 5.87 (s, 2H, CH_2_-CO), 7.41 (t, 1H, *J* = 7.2 Hz, Ar-H), 7.45 (t, 2H, *J* = 7.35 Hz, Ar-H), 7.52 (d, 2H, *J* = 7.26 Hz, Ar-H), 7.68–7.71 (m, 2H, Ar-H), 7.85 (d, 2H, *J* = 9.1 Hz, Ar-H), 8.01 (t, 1H, *J* = 9.2 Hz, Ar-H), 8.07 (d, 1H, *J* = 7.8 Hz, Ar-H), 8.28 (d, 2H, *J* = 9.18 Hz, Ar-H), 9.91 (s, 1H, NCHN), 11.34 (s, 1H, NH). ^13^C-NMR (DMSO-d6, 150 MHz) δ (ppm): 49.3, 50.0, 113.8, 114.0, 119.15 (2C), 125.1 (2C), 126.8, 127, 128.2 (2C), 128.9, 129.1 (2C), 130.4, 132.0, 133.7, 142.7, 143.6, 144.2, 164.6 (C=O). MS (ESI+): *m*/*z* calcd for C_22_H_19_N_4_O_3_^+^ [M − Br]^+^ 387; found 387 [M − Br]^+^.
*1-Benzyl-3-{2-oxo-2-(o-tolylamino)ethyl}-1H-benzo[d]imidazol-3-ium bromide* (**9i**): White crystalline solid; Yield: 90%; m.p. 162–163 °C. ^1^H-NMR (DMSO-d6, 600 MHz) δ (ppm): 2.3 (s, 3H, CH_3_), 5.67 (s, 2H, CH_2_-Ph), 5.9 (s, 2H, CH_2_-CO), 7.13 (t, 1H, *J* = 7.14 Hz, Ar-H), 7.19 (t, 1H, *J* = 7.11 Hz, Ar-H), 7.2 (d, 1H, *J* = 7.32 Hz, Ar-H), 7.39 (t, 1H, *J* = 7.2 Hz, Ar-H), 7.44 (t, 3H, *J* = 7.26 Hz, Ar-H), 7.53 (d, 2H, *J* = 7.26 Hz, Ar-H), 7.66–7.73 (m, 2H, Ar-H), 8.0 (d, 1H, *J* =8.2 Hz, Ar-H), 8.07 (d, 1H, *J* = 8.28 Hz, Ar-H), 10.0 (s, 1H, NCHN), 10.14 (s, 1H, NH). ^13^C-NMR (DMSO-d6, 150 MHz) δ (ppm): 17.9, 49.0, 50.0, 113.7, 113.9, 124.9, 126.1, 128.2 (4C), 128.8 (4C), 129.0 (3C), 130.5 (2C), 131.8, 143.6, 163.6 (C=O). MS (ESI+): *m*/*z* calcd for C_23_H_22_N_3_O^+^ [M − Br]^+^ 356; found 357 [M − Br + H]^+^.
*1-Benzyl-3-{2-oxo-2-(m-tolylamino)ethyl}-1H-benzo[d]imidazol-3-ium bromide* (**9j**): White crystalline solid; Yield: 88%; m.p. 132–134 °C. ^1^H-NMR (DMSO-d6, 600 MHz) δ (ppm): 2.3 (s, 3H, CH_3_), 5.60 (s, 2H, CH_2_-Ph), 5.88 (s, 2H, CH_2_-CO), 6.92 (d, 1H, *J* = 7.6 Hz, Ar-H), 7.22 (t, 1H, *J* = 7.8 Hz, Ar-H), 7.39–7.40 (m, 2H, Ar-H), 7.42–7.46 (m, 3H, Ar-H), 7.53 (d, 2H, *J* = 7.3 Hz, Ar-H), 7.65–7.71 (m, 2H, Ar-H), 7.99 (d, 1H, *J* = 7.98 Hz, Ar-H), 8.04 (d, 1H, *J* = 8.04 Hz, Ar-H), 9.99 (s, 1H, NCHN), 10.74 (s, 1H, NH). ^13^C-NMR (DMSO-d6, 150 MHz) δ (ppm): 21.1, 49.2, 50.0, 113.8, 116.5, 119.8, 124.7, 126.8, 127.0, 128.2 (3C), 129.0 (3C), 130.4, 132.0, 133.7, 137.1, 138.0, 138.2, 143.5, 163.3 (C=O). MS (ESI+): *m*/*z* calcd for C_23_H_22_N_3_O^+^ [M − Br]^+^ 356; found 356 [M − Br]^+^.
*1-Benzyl-3-{2-oxo-2-(p-tolylamino)ethyl}-1H-benzo[d]imidazol-3-ium bromide* (**9k**): White crystalline solid; Yield: 90%; m.p. 148–149 °C. ^1^H-NMR (DMSO-d6, 600 MHz) δ (ppm): 2.26 (3H, s, CH_3_), 5.59 (s, 2H, CH_2_-Ph), 5.88 (s, 2H, CH_2_-CO), 7.16 (d, 2H, *J* = 8.34 Hz, Ar-H), 7.39–7.40 (m, 1H, Ar-H), 7.45 (t, 2H, *J* = 6 Hz, Ar-H), 7.52 (dd, 4H, *J* = 7.8, 16.1 Hz, Ar-H), 7.66–7.71 (m, 2H, Ar-H), 8.03 (d, 1H, *J* = 7.32 Hz, Ar-H), 8.06 (d, 1H, *J* = 7.38 Hz, Ar-H), 9.99 (s, 1H, NCHN), 10.72 (s, 1H, NH). ^13^C-NMR (DMSO-d6, 150 MHz) δ (ppm): 20.4, 49.1, 49.9, 113.8 (2C), 119.3 (3C); 126.9, 128.2 (4C); 128.8 (5C), 129.0, 129.3, 133.7, 143.5, 163.1 (C=O). MS (ESI+): *m*/*z* calcd for C_23_H_22_N_3_O^+^ [M − Br]^+^ 356; found 357 [M − Br + H]^+^.
*1-Benzyl-3-[2-{(2-methoxyphenyl)amino}-2-oxoethyl]-1H-benzo[d]imidazol-3-ium bromide* (**9l**): White crystalline solid; Yield: 92%; m.p. 188–189 °C. ^1^H-NMR (DMSO-d6, 600 MHz) δ (ppm): 3.88 (s, 3H, OCH_3_), 5.67 (s, 2H, CH_2_-Ph), 5.87 (s, 2H, CH_2_-CO), 6.90 (t, 1H, *J* = 7.20 Hz, Ar-H), 7.09 (d, 1H, *J* = 8.04 Hz, Ar-H), 7.13 (t, 1H, *J* = 7.68 Hz, Ar-H), 7.38 (t, 1H, *J* = 7.2 Hz, Ar-H), 7.48 (t, 2H, *J* = 7.2 Hz, Ar-H), 7.52 (d, 2H, *J* = 7.38 Hz, Ar-H), 7.64–7.70 (m, 2H, Ar-H), 7.88 (d, 1H, *J* = 7.90 Hz, Ar-H), 8.00 (dd, 2H, *J* = 8.2, 15.99 Hz, Ar-H), 10.03 (s, 1H, NCHN), 10.07 (s, 1H, NH). ^13^C-NMR (DMSO-d6, 150 MHz) δ (ppm): 49.2, 49.9, 55.8, 111.4, 113.7, 113.8, 120.3, 121.9, 125.4, 126.2, 126.8, 127.0, 128.2 (2C), 128.8, 129.0 (2C), 130.4, 131.8, 133.7, 143.5, 149.8, 163.6 (C=O). HRMS (ESI+): *m*/*z* calcd for C_23_H_22_N_3_O^+^ [M − Br]^+^ 372.1707; found 372.1714 [M − Br]^+^.
*1-Benzyl-3-[2-{(4-methoxyphenyl)amino}-2-oxoethyl]-1H-benzo[d]imidazol-3-ium bromide* (**9m**): White crystalline solid; Yield: 90%; m.p. 171–173 °C. ^1^H-NMR (DMSO-d6, 600 MHz) δ (ppm): 3.71 (s, 3H, OCH_3_), 5.58 (s, 2H, CH_2_-Ph), 5.87 (s, 2H, CH_2_-CO), 6.90–6.93 (m, 2H, Ar-H), 7.37–7.39 (m, 1H, Ar-H), 7.43 (t, 2H, *J* = 14.7 Hz, Ar-H), 7.53 (dd, 4H, *J* = 3.4, 8.3 Hz, Ar-H), 7.64–7.69 (m, 2H, Ar-H), 7.99 (d, 1H, *J* = 7.92 Hz, Ar-H), 8.08 (d, 1H, *J* = 7.98 Hz, Ar-H), 10.00 (s, 1H, NCHN), 10.69 (s, 1H, NH). ^13^C-NMR (DMSO-d6, 150 MHz) δ (ppm): 49.1, 50.0, 55.2, 113.8, 114.0, 121.0 (2C), 126.8, 127, 128.2 (3C), 128.8, 129.0 (3C), 130.4, 131.2, 132.0, 133.7, 143.5, 155.70, 162.8 (C=O). MS (ESI+): *m*/*z* calcd for C_23_H_22_N_3_O^+^ [M − Br]^+^ 372; found 373 [M − Br + H]^+^.

### 3.5. General Procedure for the Preparation of Benzimidazolium Salts **10**(**a**–**m**)

1-Benzyl-2-methyl-1H-benzimidazole **7b** (0.23 g, 1 mmol) was dissolved in acetonitrile (30 mL) in a round bottom flask. The solution of 2-bromo-N-arylacetamides **8**(**a**–**m**) (1 mmol) in acetonitrile (10 mL) was added dropwise in the round bottom flask and the reaction mixture was refluxed for 12–14 h. The completion of the reaction was observed by thin-layer chromatography. Finally, the contents were cooled in an ice bath to obtain the crude solid salt which was purified by column chromatography.

*1-Benzyl-3-[2-{(3-chlorophenyl)amino}-2-oxoethyl]-2-methyl-1H-benzo[d]imidazol-3-ium bromide* (**10a**): White crystalline solid; Yield: 74%; m.p. 210–213 °C. ^1^H-NMR (DMSO-d6, 600 MHz) δ (ppm): 2.94 (s, 3H, Benzimidazole-CH_3_), 5.52 (s, 2H, CH_2_-Ph), 5.83 (s, 2H, CH_2_-CO), 7.17 (d, 1H, *J* = 9.52 Hz, Ar-H), 7.31 (d, 2H, *J* = 9.2 Hz, Ar-H), 7.35–7.41 (m, 4H, Ar-H), 7.44 (t, 1H, *J* = 9.6 Hz, Ar-H), 7.61–7.67 (m, 2H, Ar-H), 7.74 (s, 1H, Ar-H), 7.94 (d, 1H, *J* = 9.4 Hz, Ar-H), 7.89 (d, 1H, *J* = 8.84 Hz, Ar-H), 10.94 (s, 1H, NH). ^13^C-NMR (DMSO-d6, 150 MHz) δ (ppm): 11.3, 48.2, 48.7, 113.3, 113.7, 118.5, 119.6, 124.5, 127.1, 127.2, 127.6 (2C), 129.0, 129.6 (2C), 131.1, 131.2, 131.8, 133.7, 134.3, 139.8, 153.6, 164.0 (C=O). MS (ESI+): *m*/*z* calcd for C_23_H_21_ClN_3_O^+^ [M − Br]^+^ 390; found 391 [M − Br + H]^+^.
*1-Benzyl-3-[2-{(4-chlorophenyl)amino}-2-oxoethyl]-2-methyl-1H-benzo[d]imidazol-3-ium bromide* (**10b**): White crystalline solid; Yield: 74%; m.p. 178–180 °C. ^1^H-NMR: (DMSO-d6, 600 MHz) δ (ppm): 2.95 (s, 3H, Benzimidazole-CH_3_), 5.52 (s, 2H, CH_2_-Ph), 5.66 (s, 2H, CH_2_-CO), 7.33(d, 2H, *J* =17.4 Hz, Ar-H), 7.36 (t, 2H, *J* = 4.9 Hz, Ar-H), 7.39–7.42 (m, 3H, Ar-H), 7.60 (d, 2H, *J* = 8.94 Hz, Ar-H), 7.65 (q, 2H, *J_1_* = *J_2_* = 18 Hz, Ar-H), 7.95 (d, 1H, *J* =7.85 Hz, Ar-H), 7.99 (d, 1H, *J* =7.74 Hz, Ar-H), 10.88 (s, 1H, NH). ^13^C-NMR (DMSO-d6, 150 MHz) δ (ppm): 10.8, 47.7, 48.2, 112.8, 113.2, 121.1 (2C), 126.5, 127.1 (2C), 127.8, 128.5, 128.8 (2C), 129.1 (3C), 130.5, 131.3, 133.8, 136.8, 153.2, 163.3 (C=O). HRMS (ESI+): *m*/*z* calcd for C_23_H_21_ClN_3_O^+^ [M − Br]^+^ 390.1368; found 390.1374 [M − Br]^+^.
*1-Benzyl-3-[2-{(2-bromophenyl)amino)}-2-oxoethyl]-2-methyl-1H-benzo[d]imidazol-3-ium bromide* (**10c**): White crystalline solid; Yield: 76%; m.p. 252 °C. ^1^H-NMR (DMSO-d6, 600 MHz) δ (ppm): 3.0 (s, 3H, Benzimidazole-CH_3_), 5.64 (s, 2H, CH_2_-Ph), 6.0 (s, 2H, CH_2_-CO), 7.20 (t, 1H, *J* = 7.5 Hz, Ar-H), 7.32–7.42 (m, 6H, Ar-H), 7.63 (q, 2H, *J_1_* = *J_2_* = 7.5 Hz, Ar-H), 7.7 (q, 2H, *J_1_* = *J _2_*= 8.2 Hz, Ar-H), 7.98 (d, 1H, *J* = 8.2 Hz, Ar-H), 8.03 (d, 1H, *J* = 8.2 Hz, Ar-H), 10.3 (s, 1H, NH), ^13^C-NMR (DMSO-d6, 150 MHz) δ (ppm): 11.0, 47.7, 48.2, 113.0, 113.2, 113.6, 118.7, 125.5, 126.5, 126.6, 127.1 (2C), 128.5, 129.1 (2C), 130.5, 130.6, 131.4, 133.8, 139.1, 148.0, 153.2, 164.0 (C=O). MS (ESI+): *m*/*z* calcd for C_23_H_21_BrN_3_O^+^ [M − Br]^+^ 434; found 434 [M − Br]^+^.
*1-Benzyl-3-[2-{(3-bromophenyl)amino}-2-oxoethyl]-2-methyl-1H-benzo[d]imidazol-3-ium bromide* (**10d**): White crystalline solid; Yield: 80%; m.p. 242 °C. ^1^H-NMR (DMSO-d6, 600 MHz) δ (ppm): 3.0 (s, 3H, Benzimidazole-CH_3_), 5.60 (s, 2H, CH_2_-Ph), 5.90 (s, 2H, CH_2_-CO), 7.31–7.35 (m, 4H, Ar-H), 7.3 (t, 1H, *J* = 7.3 Hz, Ar-H), 7.4 (t, 2H, *J* = 7.3 Hz, Ar-H), 7.5 (d, 1H, *J* = 7.3 Hz, Ar-H), 7.6–7.7 (m, 2H, Ar-H), 8.0 (s, 1H, Ar-H), 8.1 (d, 1H, *J* = 8.05 Hz, Ar-H), 8.04 (d, 1H, *J* = 7.75 Hz, Ar-H), 11.0 (s, 1H, NH). ^13^C-NMR (DMSO-d6, 150 MHz) δ (ppm): 11.0, 47.8, 48.2, 113.0, 113.3, 118.2, 121.6, 121.8, 126.5, 126.6, 126.7, 127.1 (2C), 128.4, 129.1 (2C), 130.6, 131.0, 131.4, 134.0, 139.6, 153.2, 163.6 (C=O). MS (ESI+): *m*/*z* calcd for C_23_H_21_BrN_3_O^+^ [M − Br]^+^ 434; found 434 [M − Br + H]^+^.
*1-Benzyl-3-[2-{(4-bromophenyl)amino}-2-oxoethyl]-2-methyl-1H-benzo[d]imidazol-3-ium bromide* (**10e**): White crystalline solid; Yield: 82%; m.p. 212 °C. ^1^H-NMR (DMSO-d6, 600 MHz) δ (ppm): 2.99 (s, 3H, Benzimidazole-CH_3_), 5.66 (s, 2H, CH_2_-Ph), 5.91 (s, 2H, CH_2_-CO), 7.33–7.43 (m, 5H, Ar-H), 7.52–7.54 (m, 2H, Ar-H), 7.68–7.61 (m, 4H, Ar-H), 7.98 (d, 1H, *J* = 7.4 Hz, Ar-H), 8.11 (d, 1H, *J* = 7.3 Hz, Ar-H), 11.54 (s, 1H, NH). MS (ESI^+^): *m*/*z* calcd for C_23_H_21_BrN_3_O^+^ [M − Br]^+^ 434; found 435 [M − Br + H]^+^.
*1-Benzyl-2-methyl-3-[2-{(2-nitrophenyl)amino}-2-oxoethyl]-1H-benzo[d]imidazol-3-ium bromide* **10f**): White crystalline solid; Yield: 72%; m.p. 160–161 °C. ^1^H-NMR (DMSO-d6, 600 MHz) δ (ppm): (500 MHz, DMSO-d6) δ (ppm): 2.98 (s, 3H, Benzimidazole-CH_3_), 5.73 (s, 2H, CH_2_-Ph), 5.91 (s, 2H, CH_2_-CO), 7.31–7.34 (m, 2H, Ar-H), 7.37–7.43 (m, 3H, Ar-H), 7.62–7.66 (m, 4H, Ar-H), 7.74–7.77 (m, 1H, Ar-H), 7.97 (t, *J* = 6.7 Hz, 2H, Ar-H), 8.09 (d, *J* = 8.0 Hz, 1H, Ar-H), 11.78 (s, 1H, NH). MS (ESI+): *m*/*z* calcd for C_23_H_21_N_4_O_3_^+^ [M − Br]^+^ 401; found 402 [M − Br + H]^+^.
*1-Benzyl-2-methyl-3-[2-{(3-nitrophenyl)amino}-2-oxoethyl]-1H-benzo[d]imidazol-3-ium bromide* (**10g**): White crystalline solid; Yield: 73%; m.p. 180–182 °C. ^1^H-NMR (DMSO-d6, 600 MHz) δ (ppm): 2.98 (s, 3H, Benzimidazole-CH_3_), 5.61 (s, 2H, CH_2_-Ph), 5.90 (s, 2H, CH_2_-CO), 7.34 (d, 2H, *J* = 7.26 Hz, Ar-H), 7.37 (t, 1H, *J* = 7.2 Hz, Ar-H), 7.42 (t, 2H, *J* = 7.5 Hz, Ar-H), 7.63 (dd, 1H, *J* = 1.1, 7.32 Hz, Ar-H), 7.65–7.68 (m, 3H, Ar-H), 7.91 (d, 1H, *J* = 8.16 Hz, Ar-H), 7.95–8.0 (m, 2H, Ar-H), 8.04 (d, 1H, *J* = 7.78 Hz, Ar-H), 11.29 (s, 1H, NH). ^13^C-NMR: (DMSO-d6, 150 MHz) δ (ppm): 11.0, 47.8, 48.2, 113.0, 113.2, 113.6, 118.7, 125.5, 126.5, 126.6, 127.1 (2C), 128.5, 129.1 (2C), 130.5, 130.6, 131.4, 133.8, 139.1, 148.0, 153.2, 164.0 (C=O). MS (ESI+): *m*/*z* calcd for C_23_H_21_N_4_O_3_^+^ [M − Br]^+^ 401; found 401 [M − Br]^+^.
*1-Benzyl-2-methyl-3-[2-{(4-nitrophenyl)amino)}-2-oxoethyl]-1H-benzo[d]imidazol-3-ium bromide* (**10h**): White crystalline solid; Yield: 76%; m.p. 222–224 °C. ^1^H-NMR (DMSO-d6, 600 MHz) δ (ppm): 2.96 (s, 3H, Benzimidazole-CH_3_), 5.60 (s, 2H, CH_2_-Ph), 5.87 (s, 2H, CH_2_-CO), 7.32 (d, 2H, *J* = 7.4 Hz, Ar-H), 7.37 (t, 1H, *J* = 7.2 Hz, Ar-H), 7.41 (t, 2H, *J* = 7.2 Hz, Ar-H), 7.62–7.67 (m, 2H, Ar-H), 7.84 (d, 2H, *J* = 8.8 Hz, Ar-H), 7.96 (d, 1H, *J* = 7.9 Hz, Ar-H), 8.02 (d, 1H, *J* = 7.53 Hz, Ar-H), 8.25 (d, 2H, *J* = 9.2 Hz, Ar-H), 10.88 (s, 1H, NH). ^13^C-NMR (DMSO-d6, 150 MHz) δ (ppm):10.8, 47.9, 48.2, 113.0, 113.2, 119.4 (2C), 125.0 (2C), 126.6, 126.7, 127.1 (2C), 128.5, 129.1 (2C), 130.5, 131.3, 133.8, 143.0, 144.0, 153.2, 164.2 (C=O). MS (ESI+): *m*/*z* calcd for C_23_H_21_N_4_O_3_^+^ [M − Br]^+^ 401; found 402 [M − Br + H]^+^.
*1-Benzyl-2-methyl-3-{2-oxo-2-(o-tolylamino)ethyl}-1H-benzo[d]imidazol-3-ium bromide* (**10i**): White crystalline solid; Yield: 84%; m.p. 168–170 °C. ^1^H-NMR (DMSO-d6, 600 MHz) δ (ppm): 2.25 (s, 3H, CH_3_), 2.51 (s, 3H, Benzimidazole-CH_3_), 5.59 (s, 2H, CH_2_-Ph), 5.87 (s, 2H, CH_2_-CO), 7.11–7.19 (m, 2H, Ar-H), 7.24 (d, 1H, *J* = 8.60 Hz, Ar-H), 7.30–7.41 (m, 6H, Ar-H), 7.63.7–68 (m, 2H, Ar-H), 7.99 (q, 2H, *J_1_* = *J_2_* = 9.6 Hz, Ar-H),10.08 (s, 1H, NH). ^13^C-NMR (DMSO-d6, 150 MHz) δ (ppm): 11.3, 18.2, 48.0, 48.7, 113.2, 113.8, 125.5, 126.5, 126.6, 127.0, 127.1, 127.6 (2C), 129.0, 129.6 (2C), 131.0, 131.1, 132.0, 132.4, 134.4, 135.6, 153.6, 164.0 (C=O). MS (ESI+): HR *m*/*z* calcd for C_24_H_24_N_3_O^+^ [M − Br]^+^ 370.1914; found 370.1917 [M − Br]^+^.
*1-Benzyl-2-methyl-3-{2-oxo-2-(m-tolylamino)ethyl}-1H-benzo[d]imidazol-3-ium bromide* (**10j**): White crystalline solid; Yield: 82%; m.p. 192–194 °C. ^1^H-NMR (DMSO-d6, 600 MHz) δ (ppm): 2.26 (s, 3H, CH_3_), 2.95 (s, 3H, Benzimidazole-CH_3_), 5.51 (s, 2H, CH_2_-Ph), 5.87 (s, 2H, CH_2_-CO), 6.94 (d, 1H, *J* = 7.5 Hz, Ar-H), 7.22 (t, 1H, *J* = 7.8 Hz, Ar-H), 7.32 (d, 2H, *J* = 7.2 Hz, Ar-H), 7.36 (q, 2H, *J_1_* = *J_2_* = 7.2 Hz, Ar-H), 7.41 (t, 3H, *J* = 7.39 Hz, Ar-H), 7.62–7.67 (m, 2H, Ar-H), 7.95 (d, 1H, *J* = 8.01 Hz, Ar-H), 7.98 (d, 1H, *J* =8.01 Hz, Ar-H), 10.68 (s, 1H, NH). ^13^C-NMR (DMSO-d6, 150 MHz) δ (ppm): 10.8, 21.0, 47.7, 48.1, 112.8, 113.2, 116.6, 120.0, 125.0, 126.5, 126.6, 127.1(2C), 128.5, 128.8, 129.1 (2C), 130.6, 131.4, 133.8, 137.8, 138.3, 153.1, 163.0 (C=O). MS (ESI+): *m*/*z* calcd for C_24_H_24_N_3_O^+^ [M − Br]^+^ 370; found 370 [M − Br]^+^.
*1-Benzyl-2-methyl-3-{2-oxo-2-(p-tolylamino)ethyl}-1H-benzo[d]imidazol-3-ium bromide* (**10k**): White crystalline solid; Yield: 82%; m.p. 220–221 °C. ^1^H-NMR (DMSO-d6, 600 MHz) δ (ppm): 2.26 (s, 3H, CH_3_), 2.96 (s, 3H, Benzimidazole-CH_3_), 5.51 (s, 2H, CH_2_-Ph), 5.90 (s, 2H, CH_2_-CO), 7.15 (d, 2H, *J* = 8.3 Hz, Ar-H), 7.33 (d, 2H, *J* = 7.17 Hz, Ar-H), 7.36 (t, 1H, *J* = 7.4 Hz, Ar-H), 7.41 (t, 2H, *J* = 7.3 Hz, Ar-H), 7.48 (d, 2H, *J* = 8.41 Hz, Ar-H), 7.62–7.67 (m, 2H, Ar-H), 7.98 (d, 1H, *J* = 7.5 Hz, Ar-H), 8.03 (d, 1H, *J* = 7.5 Hz, Ar-H), 10.64 (s, 1H, NH). ^13^C-NMR (DMSO-d6, 150 MHz) δ (ppm): 10.8, 20.4, 47.8, 48.1, 112.8, 113.0, 113.3, 119.4 (2C), 127.1(2C), 128.4, 129.0 (3C), 129.3 (2C), 130.6, 131.5, 133.1, 134.0, 135.6, 153.1, 163.0 (C=O). MS (ESI^+^): *m*/*z* calcd for C_24_H_24_N_3_O^+^ [M − Br]^+^ 370; found 370 [M − Br]^+^.
*1-Benzyl-3-[2-{(2-methoxyphenyl)amino}-2-oxoethyl]-2-methyl-1H-benzo[d]imidazol-3-ium bromide* (**10l**): White crystalline solid; Yield: 84%; m.p. 244–246 °C. ^1^H-NMR (DMSO-d6, 600 MHz) δ (ppm): 2.97 (s, 3H, Benzimidazole-CH_3_), 3.91 (s, 3H, OCH_3_), 5.67 (s, 2H, CH_2_-Ph), 5.91 (s, 2H, CH_2_-CO), 6.90–7.14 (m, 3H, Ar-H), 7.35–7.42 (m, 5H, Ar-H), 7.64–7.67 (m, 2H, Ar-H), 7.92 (d, 1H, *J* = 9.05 Hz, Ar-H), 8.0 (dd, 2H, *J* = 9.27, 9.61 Hz, Ar-H) 10.0 (s, 1H, NH). ^13^C-NMR (DMSO-d6, 150 MHz) δ (ppm): 11.4, 48.4, 48.7, 56.3, 112.0, 113.3, 113.8, 120.8, 122.3, 125.8, 127.0, 127.1, 127.6 (3C), 129.0, 131.1 (3C), 132.0, 134.5, 150.2, 153.8, 164.0 (C=O). MS (ESI^+^): *m*/*z* calcd for C_24_H_24_N_3_O_2_^+^ [M − Br]^+^ 386; found 386 [M − Br]^+^.
*1-Benzyl-3-[2-{(4-methoxyphenyl)amino}-2-oxoethyl]-2-methyl-1H-benzo[d]imidazole-3-ium bromide* (**10m**): White crystalline solid; Yield: 82%; m.p. 235–238 °C. ^1^H-NMR (DMSO-d6, 600 MHz) δ (ppm): 2.96 (s, 3H, Benzimidazole-CH_3_), 3.72 (s, 3H, OCH_3_), 5.51 (s, 2H, CH_2_-Ph), 5.88 (s, 2H, CH_2_-CO), 6.92 (d, 2H, *J* = 9.06 Hz, Ar-H), 7.33 (d, 2H, *J* = 7.2 Hz, Ar-H), 7.37 (t,1H, *J* = 7.2 Hz, Ar-H), 7.41 (t, 2H, *J* = 7.3 Hz, Ar-H), 7.50 (d, 2H, *J* = 9.03 Hz, Ar-H), 7.62–7.67 (m, 2H, Ar-H), 7.96 (d, 1H, *J* = 7.93 Hz, Ar-H), 8.01 (d, 1H, *J* = 7.93 Hz, Ar-H) 10.63 (s, 1H, NH). ^13^C-NMR (DMSO-d6, 150 MHz) δ (ppm): 10.8, 47.7, 48.2, 55.2, 112.8, 113.2, 114.0 (2C), 121.1 (2C), 126.5, 126.6, 127.1 (2C), 128.5, 129.1 (2C), 130.6, 131.0, 131.4, 134.0, 153.2, 155.8, 162.6 (C=O). MS (ESI^+^): *m*/*z* calcd for C_24_H_24_N_3_O_2_^+^ [M − Br]^+^ 386; found 387 [M − Br + H]^+^.

### 3.6. In Silico α-Glucosidase Inhibition Studies

The in silico analysis of all the benzimidazolium salts along with the reference acarbose was carried out by using the MOE Ver.2014.0901 (Molecular Operating Environment, Chemical Computing Group ULC., Montreal, QC, Canada).

#### 3.6.1. Ligand Preparation

The software (ChemDraw Ultra 12 + Serial, Perkin Elmer, MA, USA) was utilized to draw the structures of all these compounds. In order to display these files in MOE, structures of these compounds were saved in the MDL file (“.sdf”). The 2D structure of reference acarbose in the SDF file was retrieved from NCBI Pubchem. Afterwards, 3D protonation and energy minimizations of compounds as well as acarbose were executed under default parameters in MOE.

#### 3.6.2. Protein Preparation

The 3D structure of *Saccharomyces cerevisiae* α-glucosidase (PDB ID: 2QMJ) was downloaded from the Protein Data Bank (http://www.rcsb.org/pdb, accessed on 10 June 2021). The binding pocket with the protein residues (Asp203, Asp542, Asp327, His600, Arg526) was selected and the interactions of the ligand (acarbose) were checked. Afterwards ligand, solvent molecules, and unknown atoms from the receptor protein were removed. The active sites from the 3D structure of the protein were determined using MOE-Site Finder Module. The active sites of the ligand consist of Asp203, Tyr299, Asp327, Ile328, Asp366, Trp406, Trp441, Asp443, Met444, Arg526, Trp539, Gly541, Asp542, Asp571, Phe575, Arg598, and His600 residues. These sites were occupied by dummy atoms and the protein was 3D-protonated and energy-minimized using default parameters in the MOE software.

#### 3.6.3. Molecular Docking

Ten conformations of all the ligands were docked into the selected site of the enzyme. The MOE-Dock module was used to access binding modes of ligands with the α-glucosidase enzyme. The parameters, placement: triangle matcher, rescoring: London dG, refinement: forcefield, retain: 10 were used. The root mean square deviation rmsd values and docking scores of their top-ranked conformations were used to study the binding modes of ligands. Results of the molecular docking are depicted in Table 1.

### 3.7. In Vitro α-Glucosidase Inhibition 

The literature protocol was used to assess α-glucosidase inhibition of synthesized compounds [45]. The solution of the α-glucosidase enzyme (obtained from *Saccharomyces cerevisiae* (Sigma-Aldrich, Munich, Germany)) was prepared in phosphate buffer (pH 6.8, 100 mM). The synthesized compounds were dissolved in dimethyl sulfoxide (DMSO). A mixture of 40 μL of the α-glucosidase enzyme (0.5 U/mL), 120 μL of phosphate buffer, and 12.5 μL of each sample was prepared in 96-well microtiter plates and incubated for 5 min at 37 °C. Afterwards, 40 μL of 5 mM *p*-nitrophenyl-α-d-glucopyranoside (Sigma-Aldrich, Munich, Germany) was poured into each well and further incubated for an additional half-hour. The absorbance at 405 nm and 37 °C was noted using a microplate reader. Acarbose (Sigma-Aldrich, Munich, Germany) and DMSO were used as reference inhibitors and negative control, respectively. The tests were conducted in triplicate and percent inhibitions were calculated using the following formula:

% Inhibition = [(A control − A sample)/A control] × 100

where ‘A’ stands for absorbance. 

The IC_50_ values of the synthesized compounds and acarbose were also determined.

## 4. Conclusions

In conclusion, two novel series of benzimidazolium salts bearing amide functionality, **9**(**a**–**m**) and **10**(**a**–**m**), were synthesized and screened for α-glucosidase inhibitory potential. The C^2^-substituted benzimidazolium derivatives **10**(**a**–**m**) emerged as excellent α-glucosidase inhibitors. The bromo-substituted compound **10d** exhibited the best activity among the series, with an IC_50_ value of 14 + 0.013 µM which is 4-fold higher than the standard drug. Moreover, molecular docking studies of the most active compounds were carried out to find the interaction mode and structure-activity relationship. This study, being pioneering in its nature, will possibly be a landmark for further research for the discovery of safer drugs for the treatment of diabetic mellitus. 

## Figures and Tables

**Table 1:** In silico screening results of benzimidazolium salts **9a**–**m** and **10a**–**m**.

Compound	10a	10d	10g	10h	10l	10m	Acarbose
Docking Score (Kcal/mol)	−10.87	−10.85	−11.83	−10.94	−13.5	−13.43	−16.18
Rmsd Value (Å)	1.75	1.49	1.59	1.52	1.08	1.74	2
Interacting Residues	Asp203, Asp327	Asp327, Asp203	Asp203	Asp203, Asp542	Asp542, Asp443,	Asp203, Asp542	His600, Asp542, Arg526, Asp327, Met444, Lys480

**Table 2:** In vitro α-glucosidase inhibitory (IC_50_) activities of synthesized benzimidazolium salts.

Sr No	Code	R	R_1_	IC_50_ (µM)
1	**9a**	H	3-Cl	150.7 ± 0.045
2	**9b**	H	4-Cl	169.16 ± 0.023
3	**9c**	H	2-Br	70.01 ± 0.037
4	**9d**	H	3-Br	171.11± 0.089
5	**9e**	H	4-Br	198 ± 0.103
6	**9f**	H	2-NO_2_	241 ± 0.027
7	**9g**	H	3-NO_2_	215.73 ± 0.112
8	**9h**	H	4-NO_2_	370 ± 0.032
9	**9i**	H	2-CH_3_	259.86 ± 0.043
10	**9j**	H	3-CH_3_	200.14 ± 0.039
11	**9k**	H	4-CH_3_	201.13 ± 0.105
12	**9l**	H	2-OCH_3_	74.94 ± 0.028
13	**9m**	H	4-OCH_3_	279.28 ± 0.030
14	**10a**	CH_3_	3-Cl	22 ± 0.040
15	**10b**	CH_3_	4-Cl	60 ± 0.041
16	**10c**	CH_3_	2-Br	46 ± 0.106
17	**10d**	CH_3_	3-Br	14 ± 0.013
18	**10e**	CH_3_	4-Br	30 ± 0.049
19	**10f**	CH_3_	2-NO_2_	60 ± 0.025
20	**10g**	CH_3_	3-NO_2_	19 ± 0.086
21	**10h**	CH_3_	4-NO_2_	17 ± 0.067
22	**10i**	CH_3_	2-CH_3_	100 ± 0.048
23	**10j**	CH_3_	3-CH_3_	60 ± 0.041
24	**10k**	CH_3_	4-CH_3_	32.27 ± 0.050
25	**10l**	CH_3_	2-OCH_3_	25± 0.011
26	**10m**	CH_3_	4-OCH_3_	16.16 ± 0.026
	Acarbose	-	-	58.8 ± 0.015

## Data Availability

The data presented in this study are available in the Supplementary Materials.

## Supplementary Materials

The following are available online: ^1^H-NMR, ^13^C-NMR, and MS spectra of the synthesized compounds having potent enzyme inhibition activities.

## Sample Availability

Samples of the compounds are available from the authors.
