# Supplementary material for: Discovery of Amide-Functionalized Benzimidazolium Salts as Potent α-Glucosidase Inhibitors
Source: Molecules. 2021 Aug 6;26(16):4760. doi: 10.3390/molecules26164760 (PMC8400806; doi:10.3390/molecules26164760)
Supplement: Supplementary file 1 [file molecules-26-04760-s001.zip › molecules-1300367-supplementary.pdf]

Discovery of amide-functionalized benzimidazolium salts as potent α-glucosidase Inhibitors

Imran Ahmad Khan [1], Matloob Ahmad [1,*], Usman Ali Ashfaq[2], Sadia Sultan [3,4,*] and Magdi E. A. Zaki [5,*]

Characterization Data of Potent Compounds

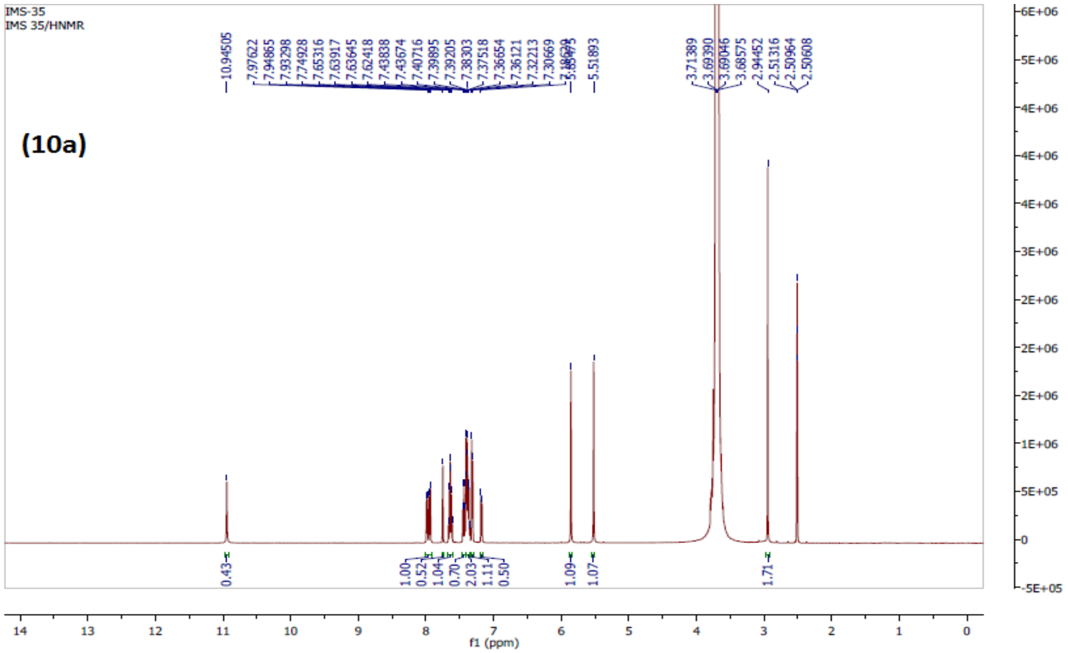

Figure S1. ^{1}H NMR Spectra of compound **10a**

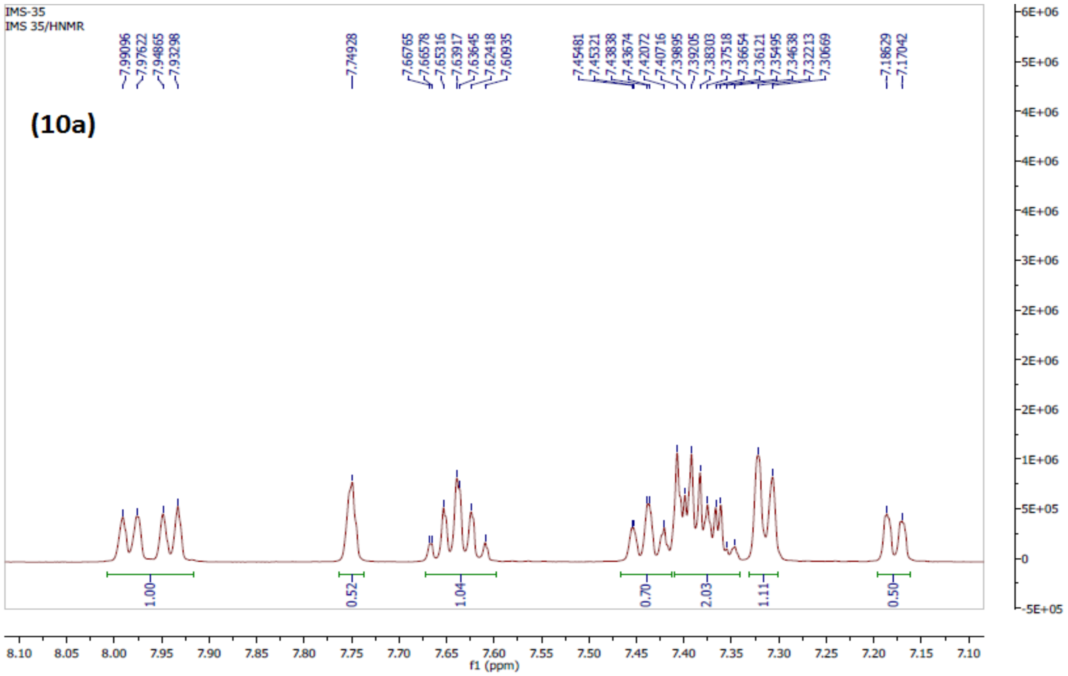

Figure S1a. Expanded version of ^{1}H NMR Spectra of compound **10a**

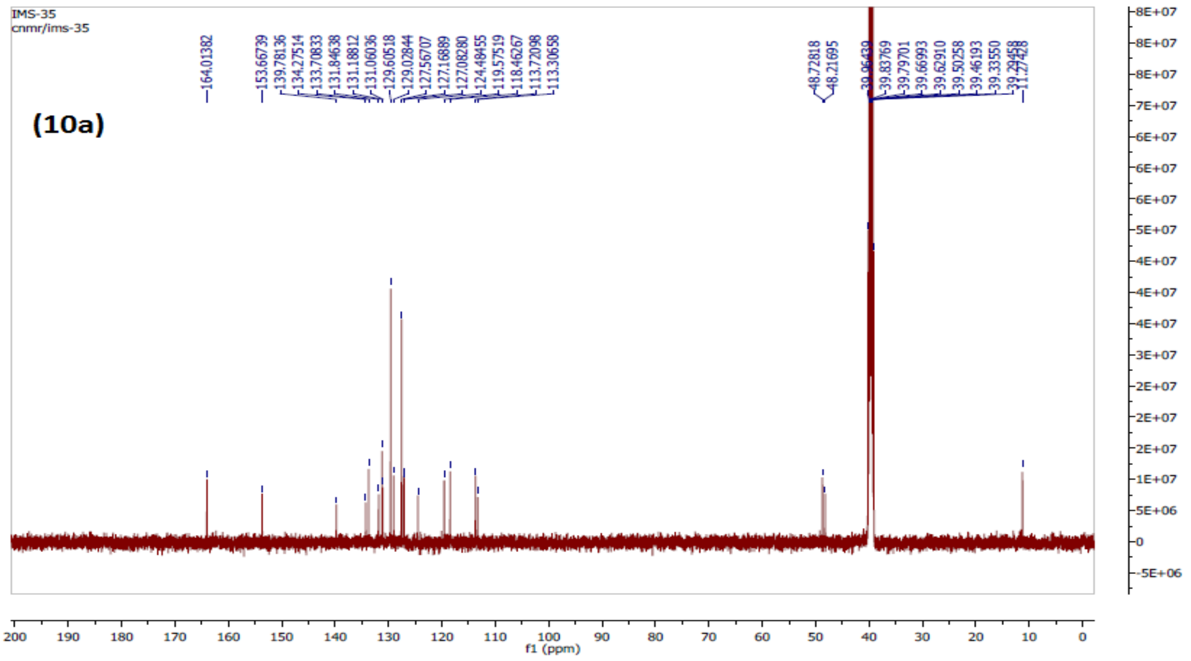

Figure S2. ^{13}C NMR Spectra of compound **10a**

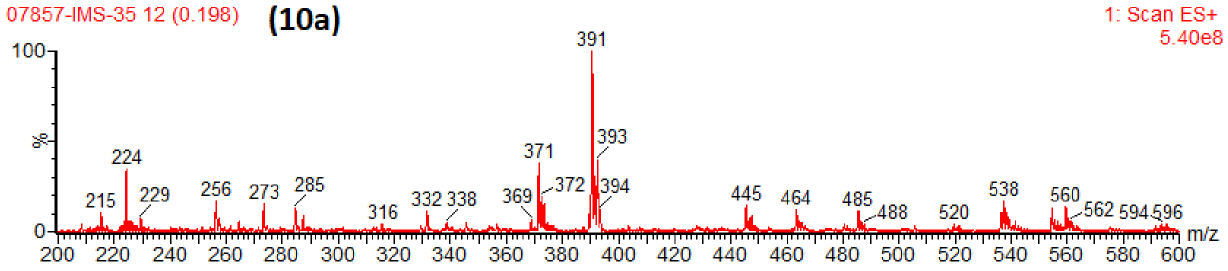

Figure S3. MS (ESI+) Spectra of compound **10a**

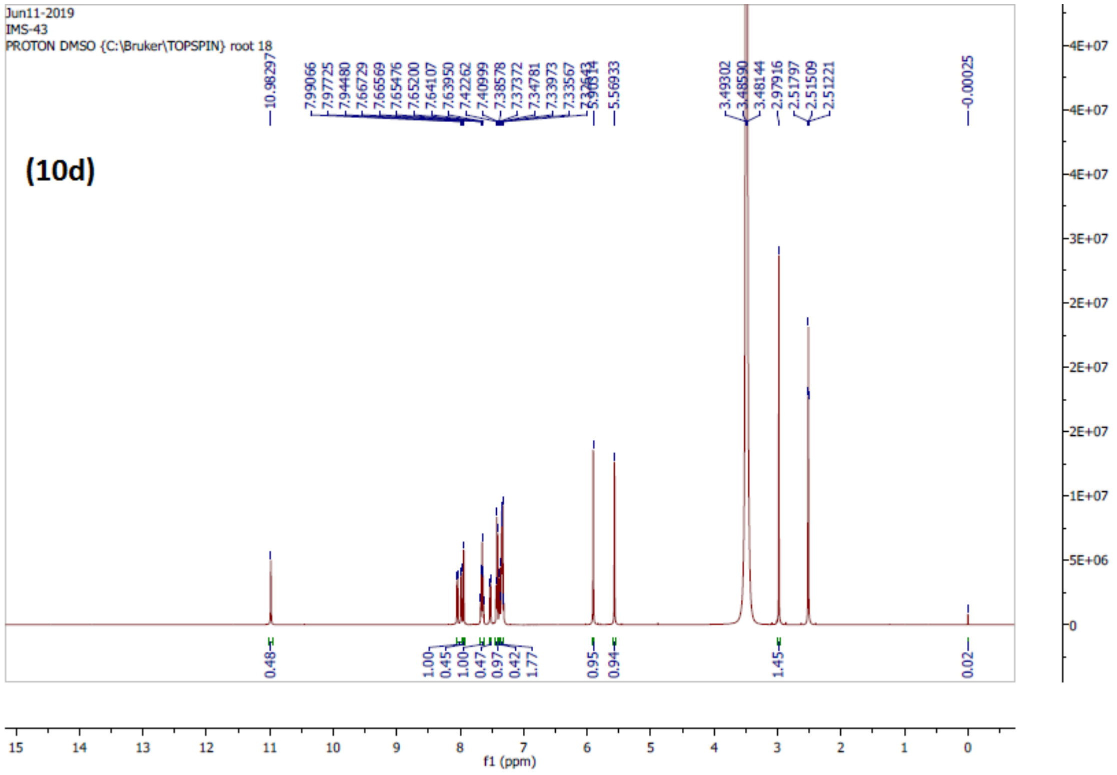

Figure S4. ^{1}H NMR Spectra of compound **10d**

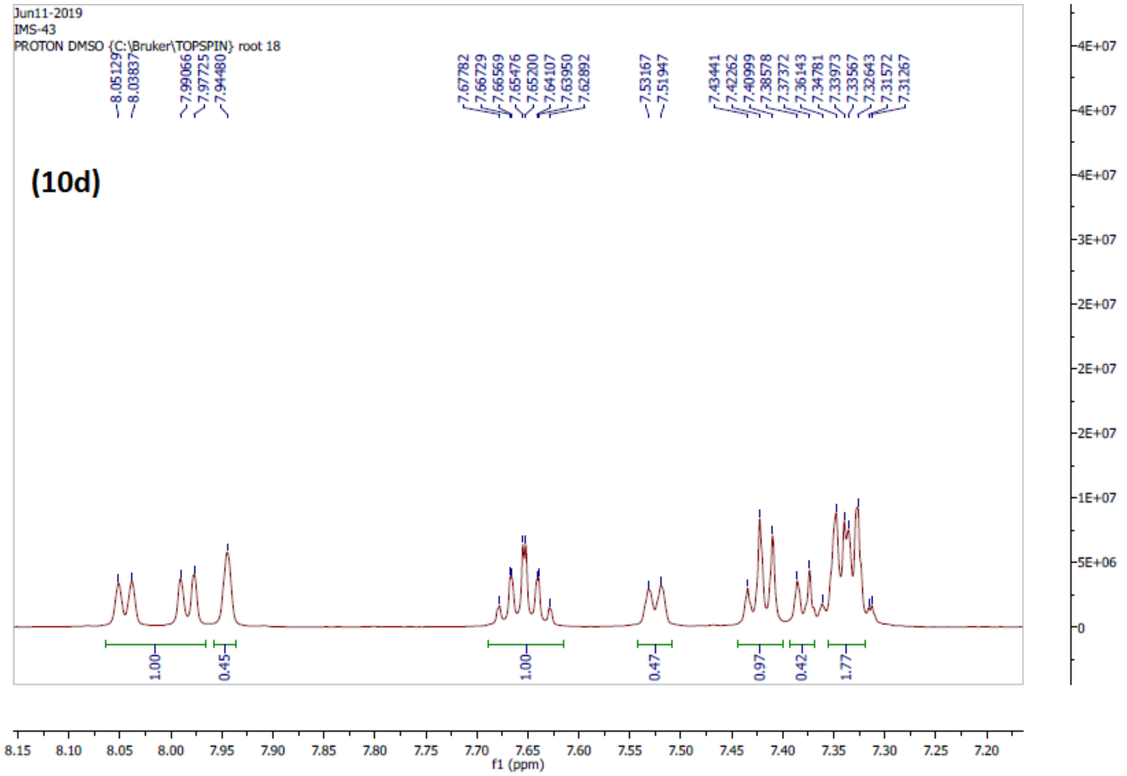

Figure S4a. Expanded version of ^{1}H NMR Spectra of compound **10d**

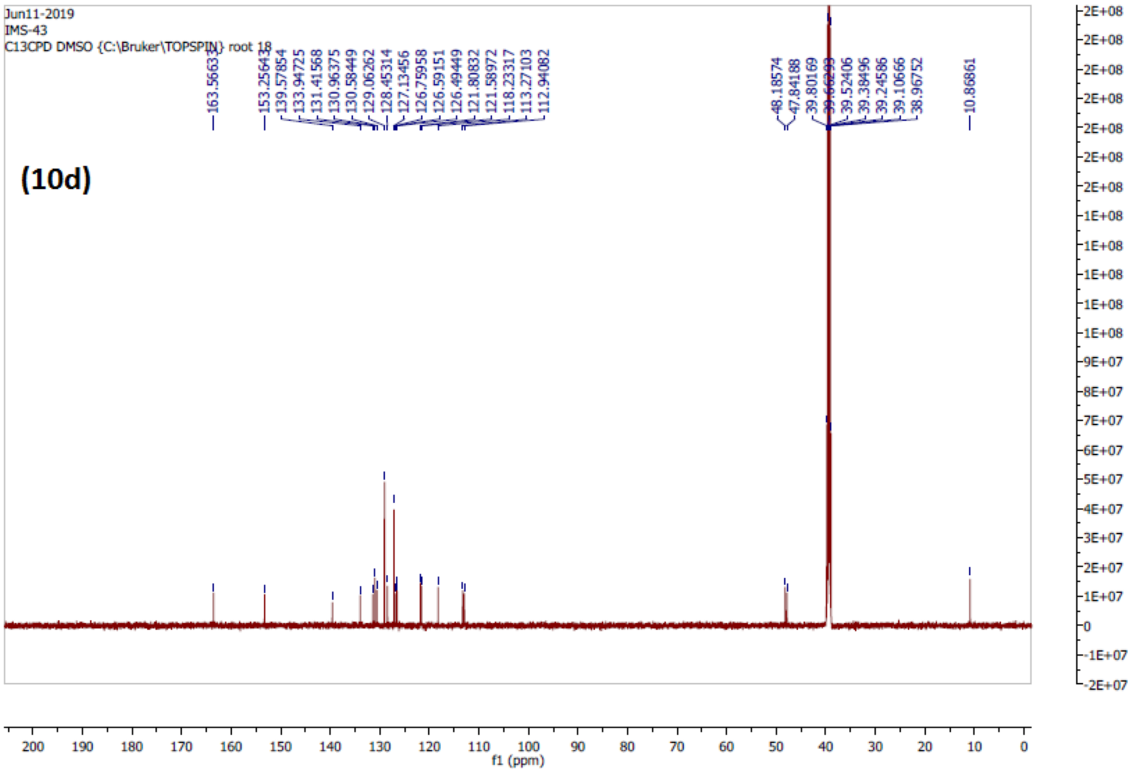

Figure S5. ^{13}C NMR Spectra of compound **10d**

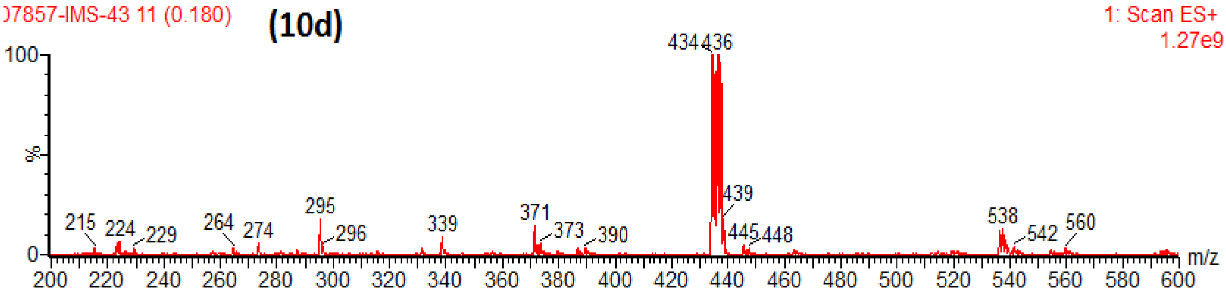

Figure S6. MS (ESI+) Spectra of compound **10d**

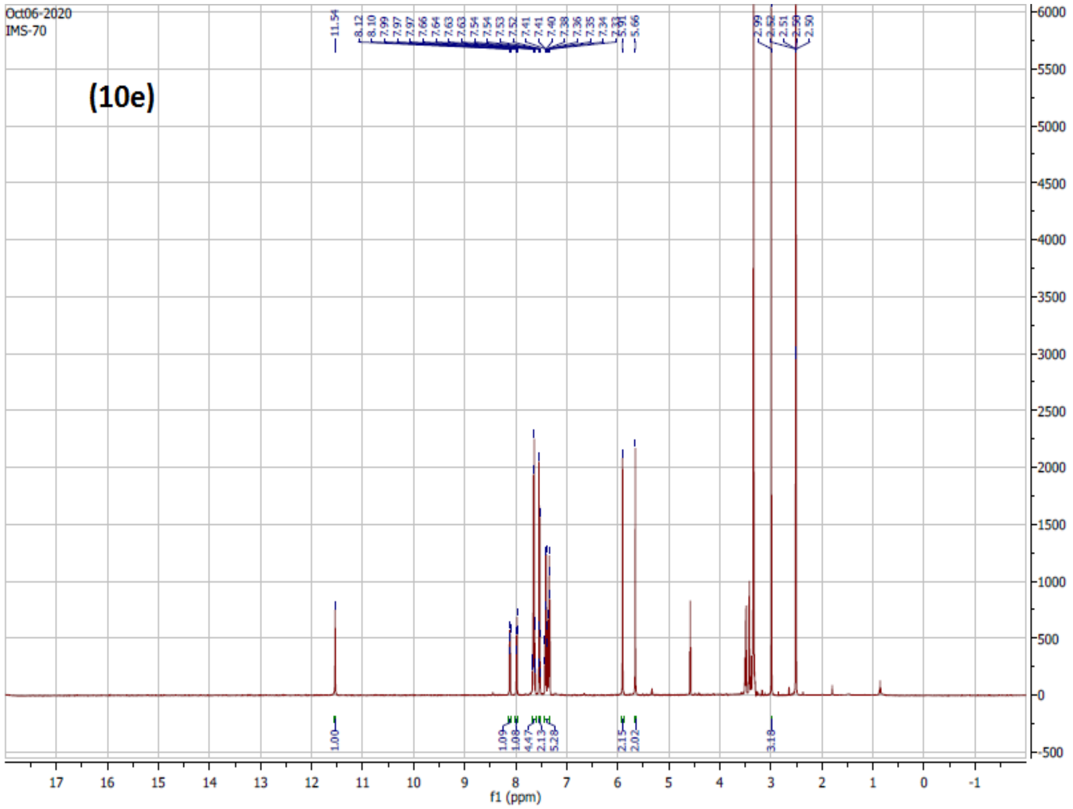

Figure S7. [1]*H* NMR Spectra of compound **10e**

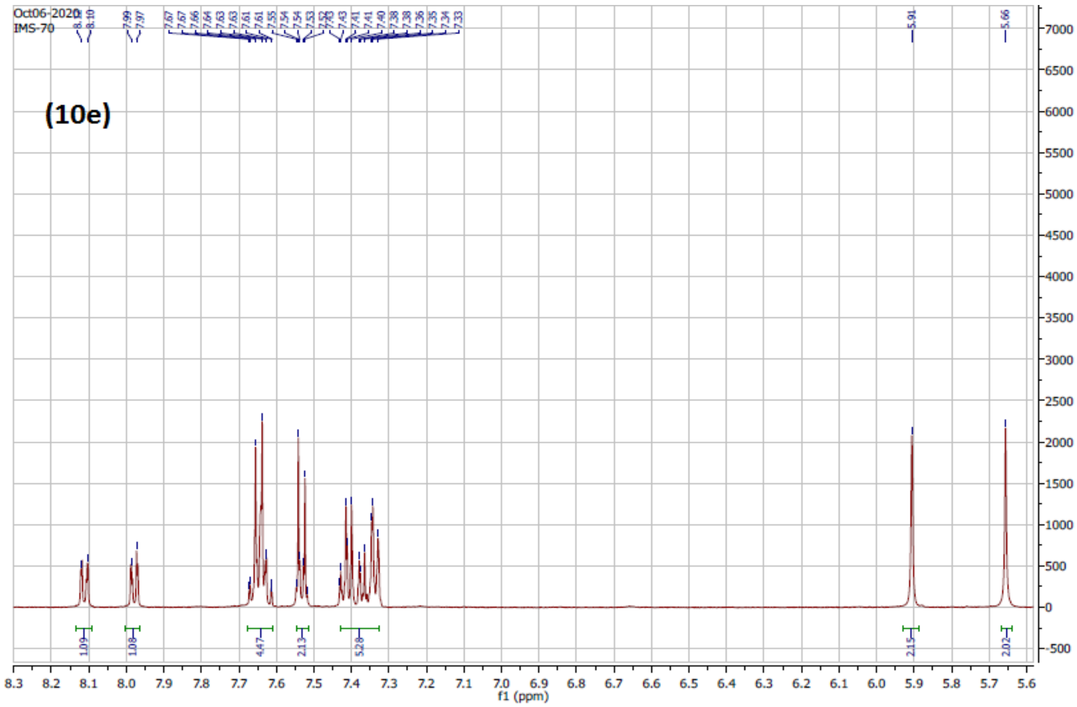

Figure S7a. Expanded version of [1]*H* NMR Spectra of compound **10e**

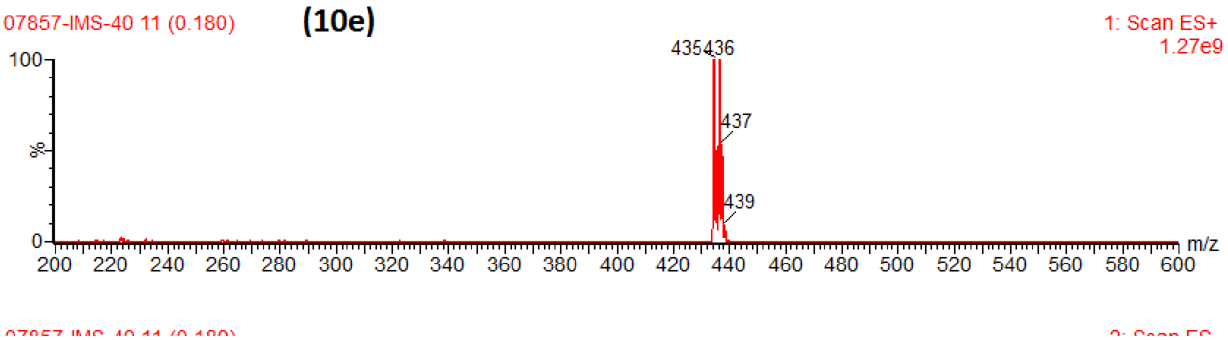

Figure S8. MS (ESI+) Spectra of compound **10e**

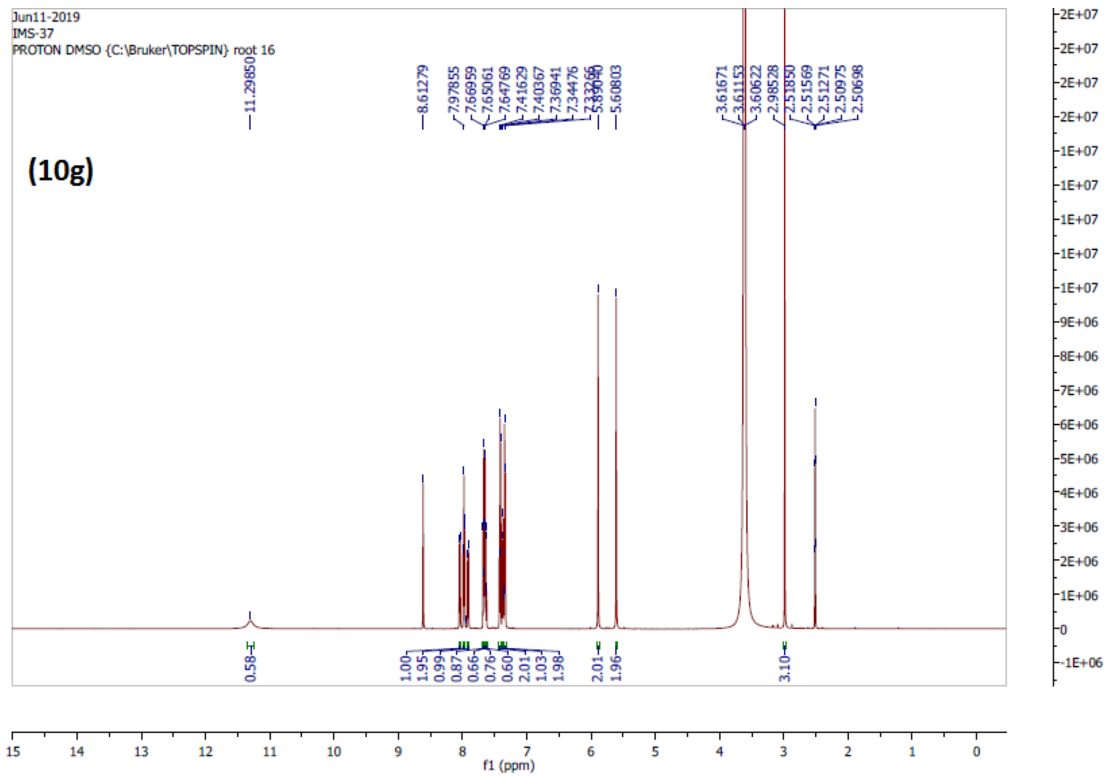

Figure S9. ^{1}H NMR Spectra of compound **10g**

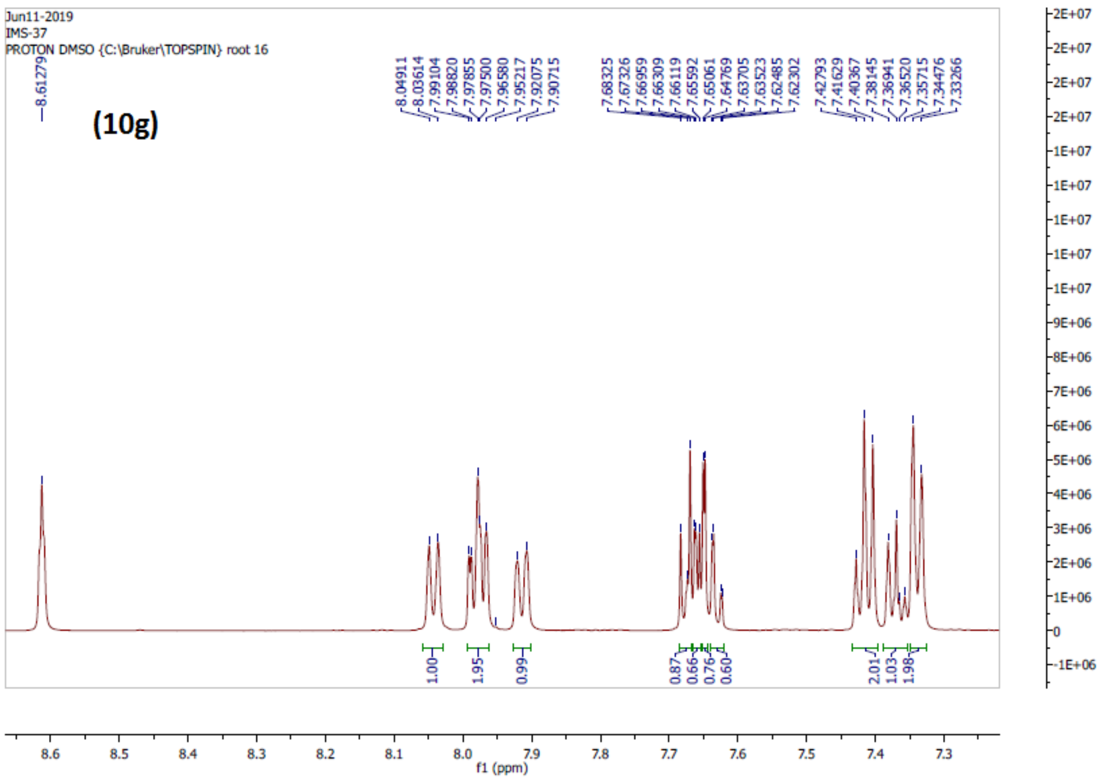

Figure S9a. Expanded version of ^{1}H NMR Spectra of compound **10g**

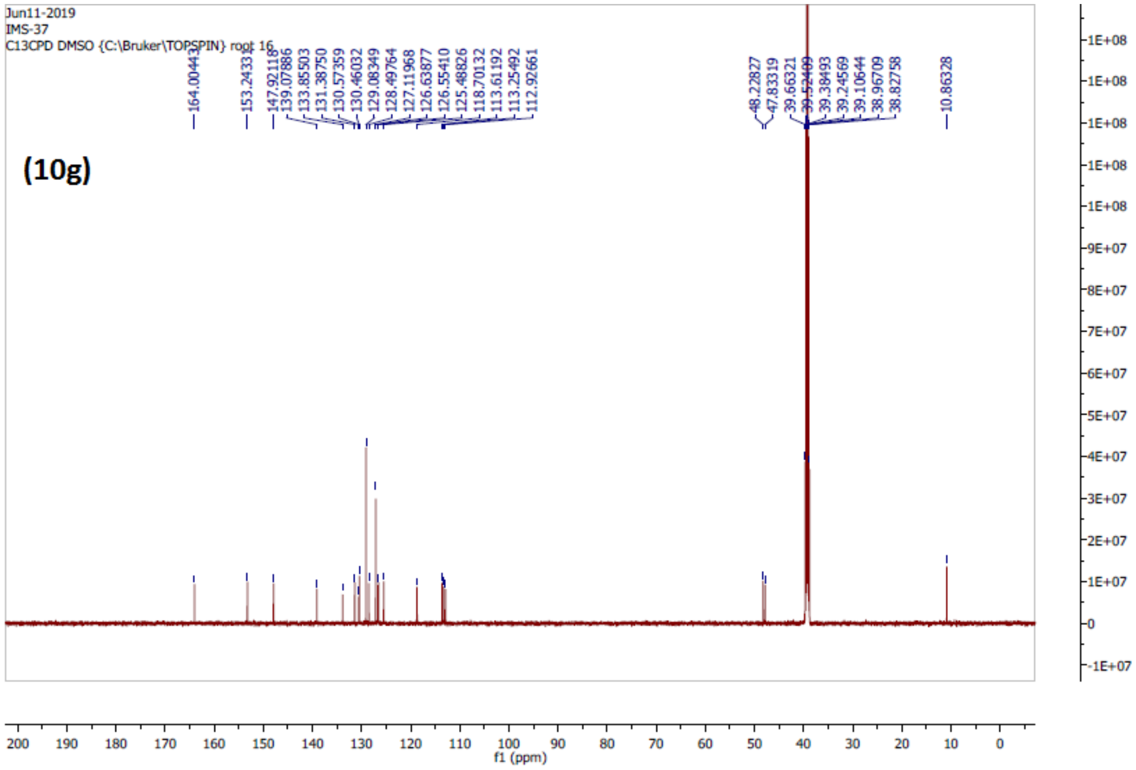

Figure S10. ^{13}C NMR Spectra of compound **10g**

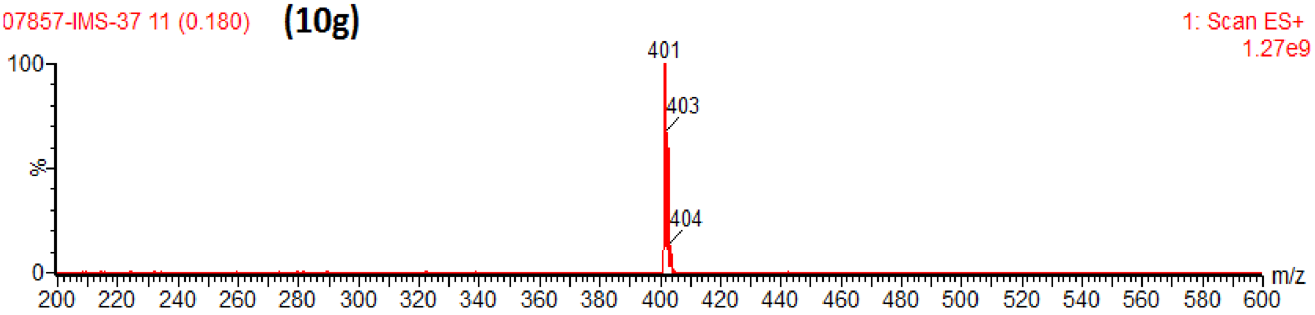

Figure S11. MS (ESI+) Spectra of compound **10g**

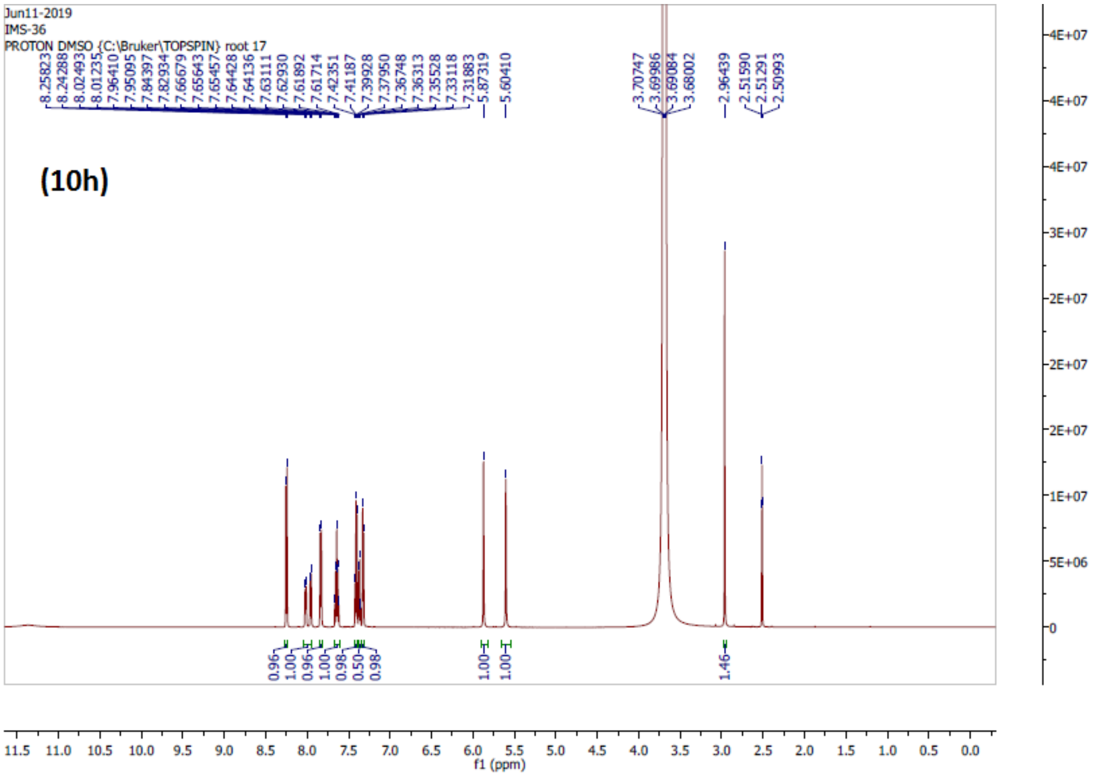

Figure S12. [1]*H* NMR Spectra of compound **10h**

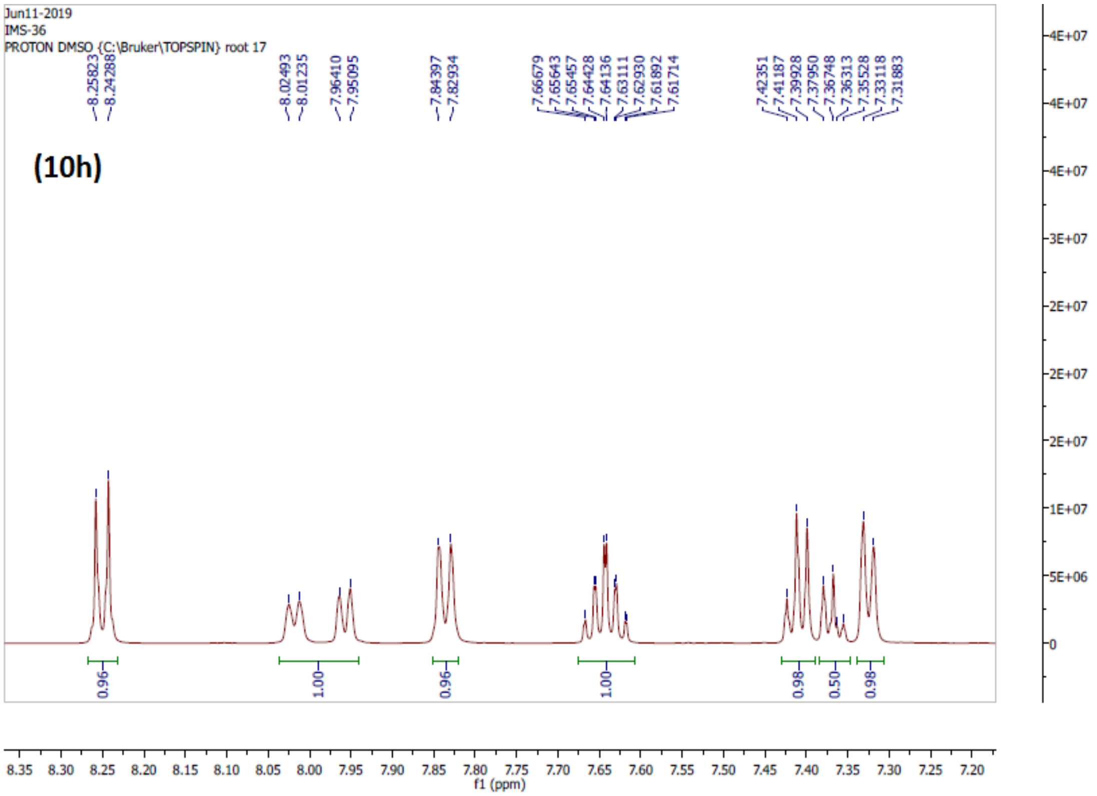

Figure S12a. Expanded version of [1]*H* NMR Spectra of compound **10h**

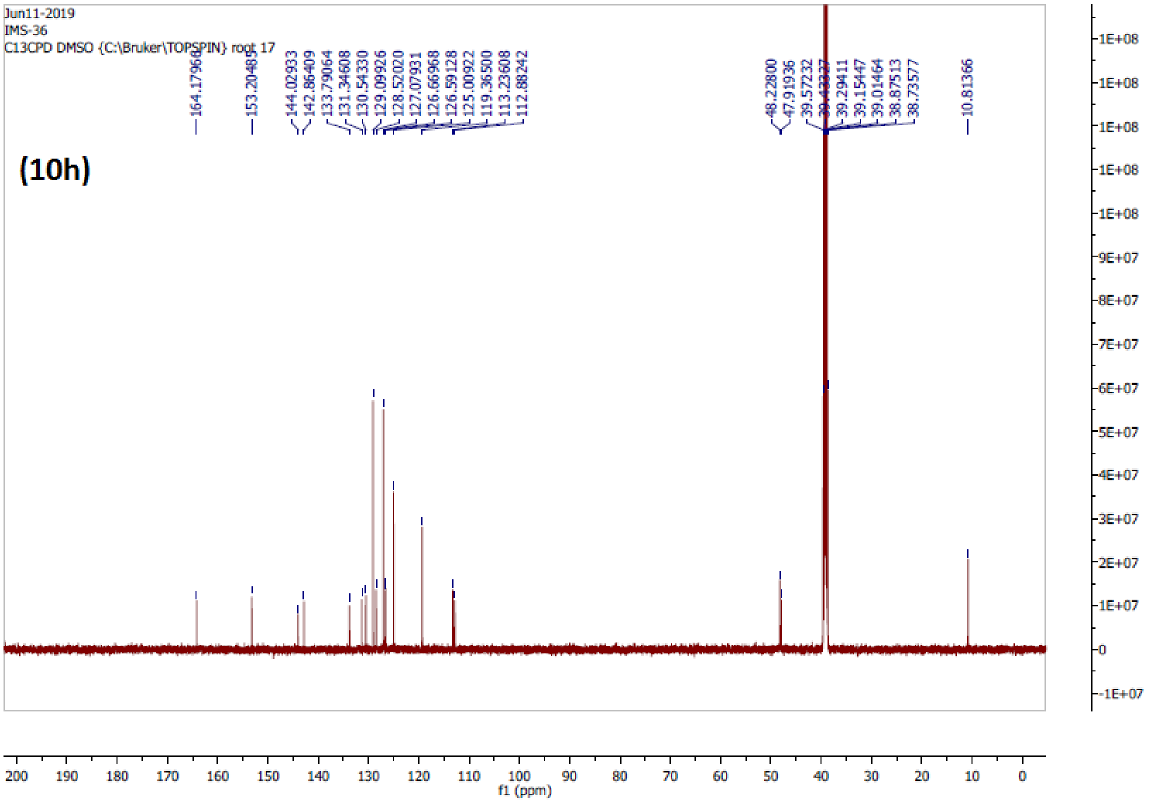

Figure S13. ^{13}C NMR Spectra of compound **10h**

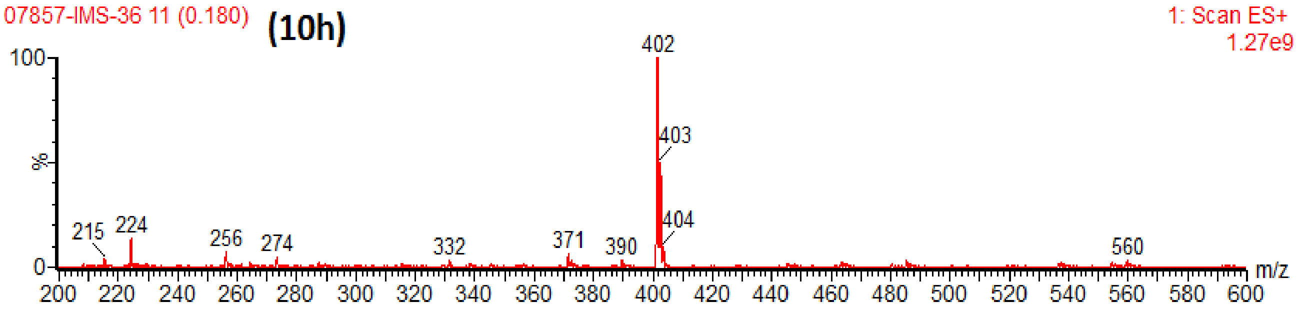

Figure S14. MS (ESI+) Spectra of compound **10h**

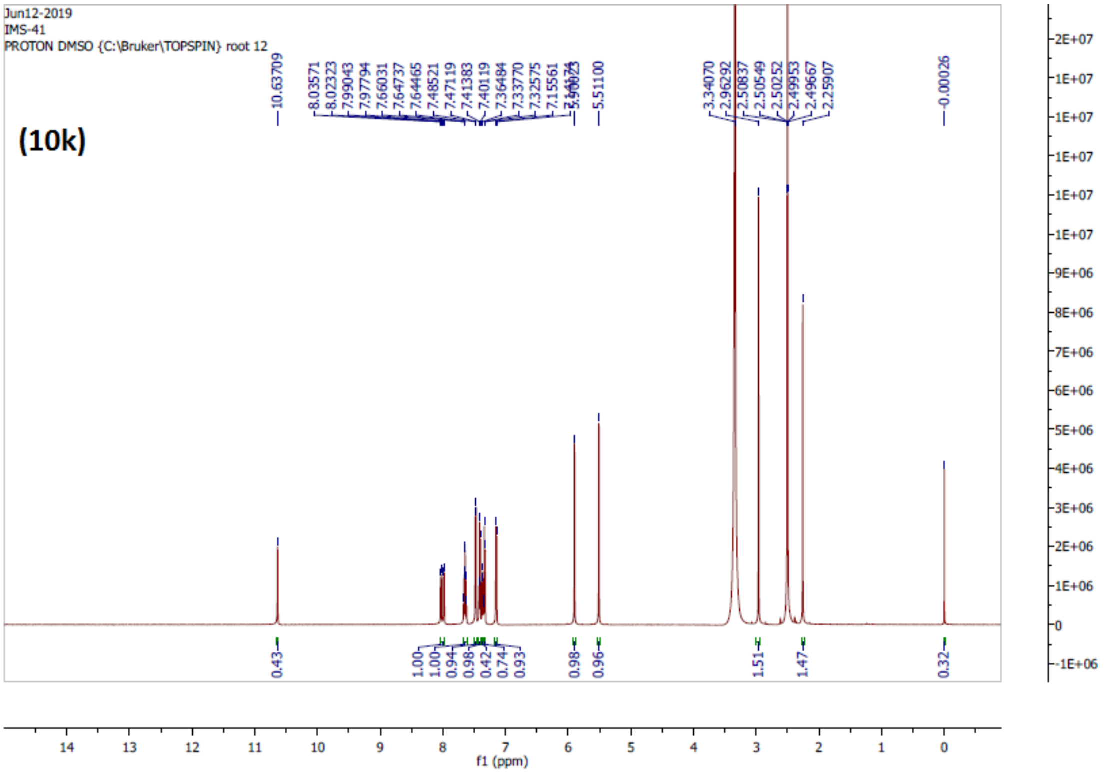

Figure S15. ^{1}H NMR Spectra of compound **10k**

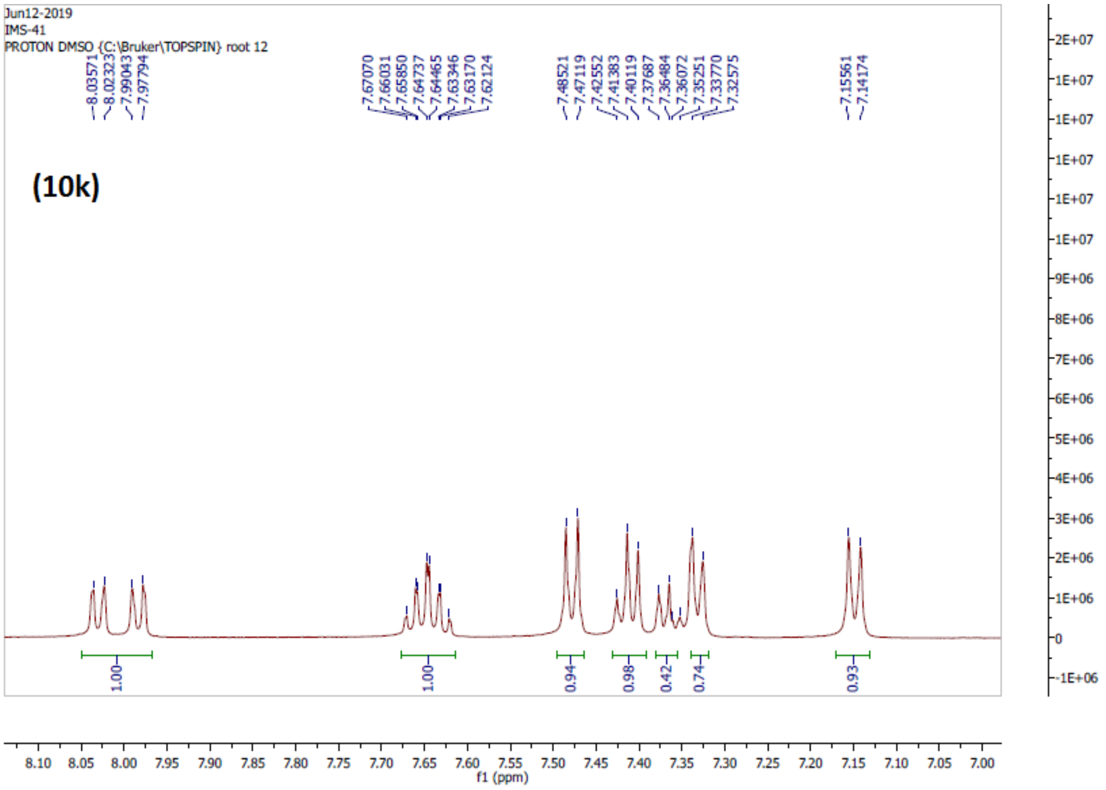

Figure S15a. Expanded version of ^{1}H NMR Spectra of compound **10k**

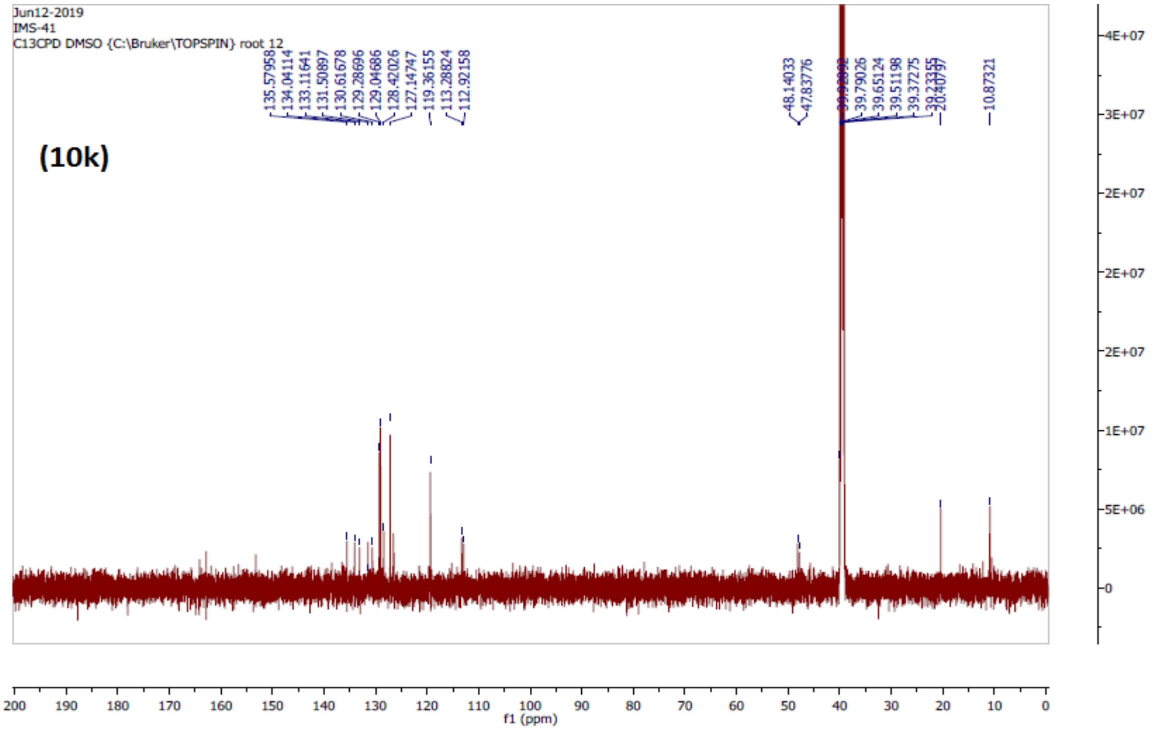

Figure S16. ^{13}C NMR Spectra of compound **10k**

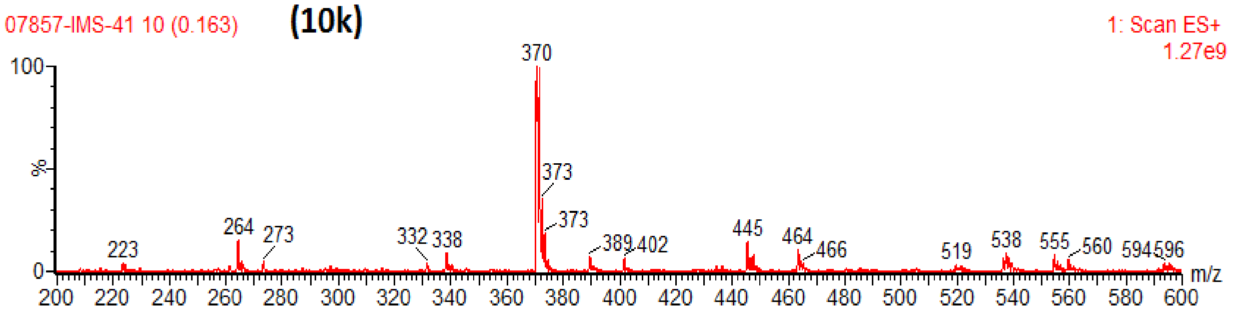

Figure S17. MS (ESI+) Spectra of compound **10k**

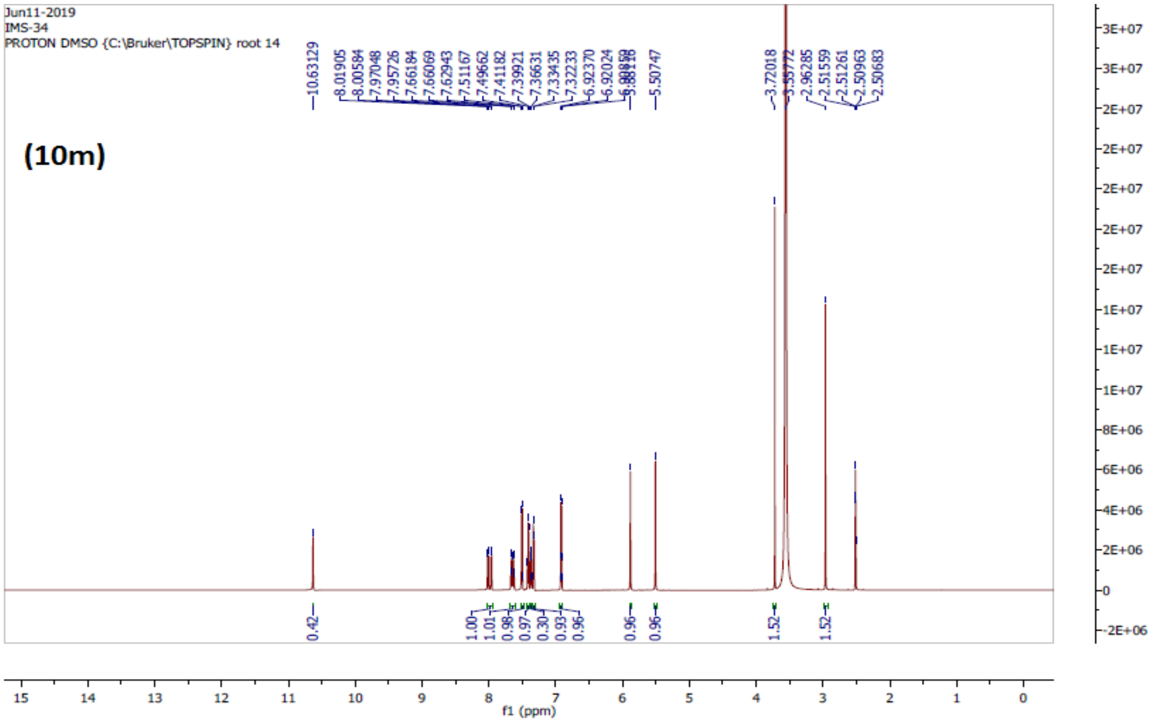

Figure S18. ^{1}H NMR Spectra of compound **10m**

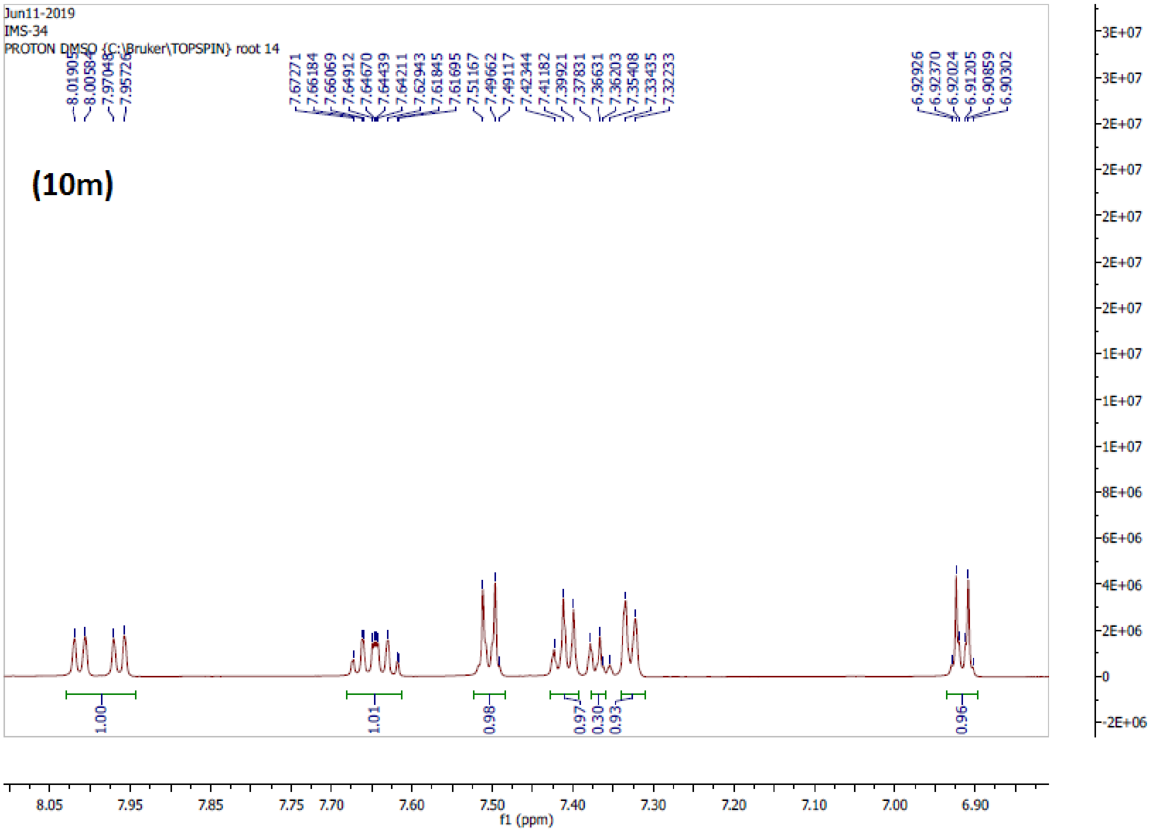

Figure S18a. Expanded version of ^{1}H NMR Spectra of compound **10m**

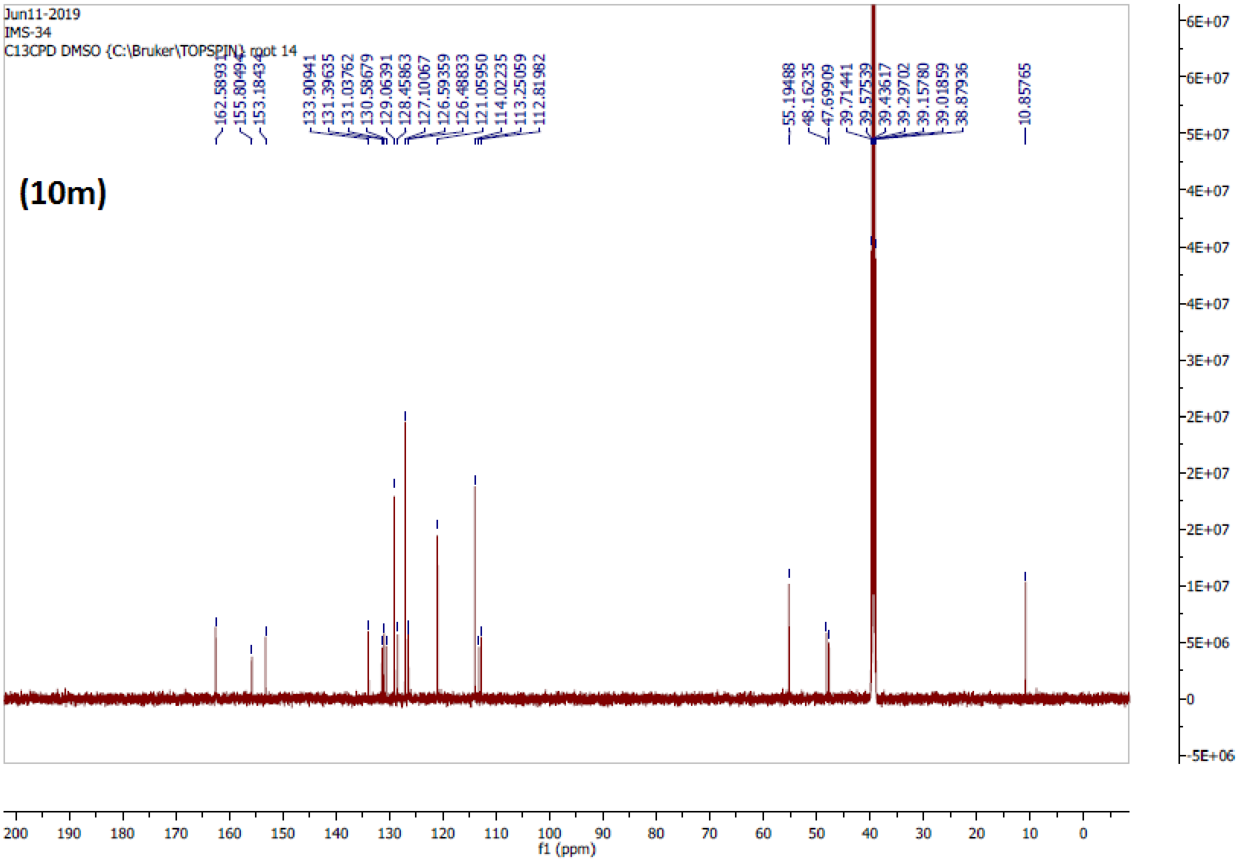

Figure S19. ^{13}C NMR Spectra of compound **10m**

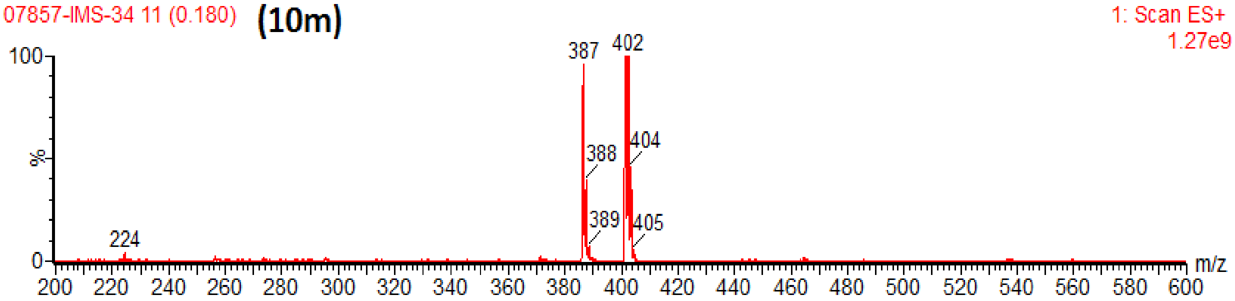

Figure S20. MS (ESI+) Spectra of compound **10m**
